# Urinary MicroRNA Profiling Predicts the Development of Microalbuminuria in Patients with Type 1 Diabetes

**DOI:** 10.3390/jcm4071498

**Published:** 2015-07-17

**Authors:** Christos Argyropoulos, Kai Wang, Jose Bernardo, Demetrius Ellis, Trevor Orchard, David Galas, John P. Johnson

**Affiliations:** 1 Department of Medicine, Division of Nephrology, University of New Mexico, 901 University Blvd SE, Albuquerque, NM 87106, USA; E-Mail: cargyropoulos@salud.unm.edu; 2 Institute for Systems Biology, 401 Terry Ave. North, Seattle, WA 98109, USA; E-Mail: kai.wang@systemsbiology.org; 3 Department of Medicine, Renal and Electrolyte Division, University of Pittsburgh, 3550 Terrace Street, Pittsburgh, PA 15261, USA; E-Mail: jfb15@pitt.edu; 4 Children’s Hospital of Pittsburgh, One Children’s Hospital Drive 4401 Penn Avenue, Pittsburgh, PA 15224, USA; E-Mail: ellisd@upmc.edu; 5 Department of Epidemiology, Graduate School of Public Health, University of Pittsburgh, 130 DeSoto Street, Pittsburgh, PA 15261, USA; E-Mail: orchardt@edc.pitt.edu; 6 Pacific Northwest Diabetes Research Institute, 720 Broadway, Seattle, WA 98103, USA; E-Mail: djgalas@gmail.com

**Keywords:** microRNAs, microalbuminuria, Type 1 diabetes, gene ontology, target analysis, prognostic model

## Abstract

Microalbuminuria provides the earliest clinical marker of diabetic nephropathy among patients with Type 1 diabetes, yet it lacks sensitivity and specificity for early histological manifestations of disease. In recent years microRNAs have emerged as potential mediators in the pathogenesis of diabetes complications, suggesting a possible role in the diagnosis of early stage disease. We used quantiative polymerase chain reaction (qPCR) to evaluate the expression profile of 723 unique microRNAs in the normoalbuminuric urine of patients who did not develop nephropathy (*n* = 10) relative to patients who subsequently developed microalbuminuria (*n* = 17). Eighteen microRNAs were strongly associated with the subsequent development of microalbuminuria, while 15 microRNAs exhibited gender-related differences in expression. The predicted targets of these microRNAs map to biological pathways known to be involved in the pathogenesis and progression of diabetic renal disease. A microRNA signature (miR-105-3p, miR-1972, miR-28-3p, miR-30b-3p, miR-363-3p, miR-424-5p, miR-486-5p, miR-495, miR-548o-3p and for women miR-192-5p, miR-720) achieved high internal validity (cross-validated misclassification rate of 11.1%) for the future development of microalbuminuria in this dataset. Weighting microRNA measurements by their number of kidney-relevant targets improved the prognostic performance of the miRNA signature (cross-validated misclassification rate of 7.4%). Future studies are needed to corroborate these early observations in larger cohorts.

## 1. Introduction

Diabetic nephropathy is a major contributor to the heightened morbidity and mortality of patients with Type 1 diabetes [1]. Microalbuminuria testing is the current standard for diagnosing early renal involvement, yet urine albumin testing is not ideal as it is affected by many common clinical factors, e.g., acute febrile disease, exercise and transient loss of glycemic control [2]. Furthermore, severe non-Kimmelstein-Wilson structural lesions (e.g., mesangial expansion, glomerulotubular junction abnormalities or even tubulointerstitial lesions) [3,4,5,6,7] may already be evident before the development of microalbuminuria, highlighting the need for more sensitive, non-invasive biomarkers of early stage diabetic nephropathy [1,8,9]. In patients with diabetes, the risk of increased cardiovascular morbidity, mortality and further deterioration of their renal function varies with both the degree of renal impairment (estimated Glomerular Filtration Rate, eGFR) and proteinuria [10,11,12]. Understanding the biological correlates of microalbuminuria may thus shed some light on the heightened cardiac and renal risk of patients with diabetes.

In recent years it has become apparent that gene expression is post-transcriptionally controlled by microRNAs, short (21–23 nt) non-coding RNAs which bind to the 3′-untranslated region of specific mRNAs. MicroRNAs appear to be a promising biomarker by virtue of their modulatory pathobiological role [13], high expression in urine and their stability under storage conditions [14]. Experimental work has implicated microRNAs in the pathogenesis of renal disease [15,16,17] and diabetic nephropathy [18]. Previous research [19,20,21] has highlighted associations between microRNA expression changes in patients with Type 1 or 2 diabetes. Using a case-control design, we reported the urinary miRNA profiles of Type 1 diabetics in groups matched by age, gender, duration of disease and glycated hemoglobin (HbA1C) without and with renal disease and intermittent and persistent microalbuminuria [22]. We have shown that urinary miRNA profiles vary significantly across the different stages of diabetic nephropathy and map to overlapping target protein pathways known to be targeted in diabetic kidney disease.

Since the appearance of microalbuminuria is widely taken as the initial clinical manifestation of diabetic nephropathy, we decided to undertake a secondary analysis of our publicly available microRNA expression dataset [22] to explore the hypothesis that microRNAs may be useful in the early identification of patients who will go on to develop microalbuminuria. We thus compared the urinary microRNA profiles in normoalbuminuric samples from individuals who never developed signs of nephropathy and others who subsequently developed microalbuminuria over a period of 18 years. We also examined associations between microRNA expression and gender in patients with Type 1 diabetes. Gender has been found to be an important modifier in the risk for adverse renal outcomes in many renal diseases, but the protective effect of gender appears to be lost in diabetic nephropathy. Subsequently, we developed a urinary microRNA classification rule for the future development of microalbuminuria in normoalbuminuric patients with Type 1 diabetes. miRNA components of this signature were validated in an independent cohort of patients with variable degrees of microalbuminuria and Type 1 diabetes [20], but also in another study of patients with untreated hypertensive kidney disease [23].

## 2. Experimental Section

### 2.1. Patients and Samples

A full description of the methods for collection of samples and identification of participants are available in our previous publication [22] and only a brief summary will be given here. Urinary microRNAs were profiled in participants of the Pittsburgh Epidemiology of Diabetes Complications (EDC) study, a historical prospective cohort of patients with Type 1 diabetes [24]. In EDC, patients were eligible for enrollment if they were diagnosed with Type 1 diabetes from 1950–1980 at the Children’s Hospital of Pittsburgh. A total of 906 patients, out of 1124 eligible for participation, agreed to participate in the EDC. Baseline examinations of the EDC were undertaken between 1986 and 1988 and patients were subsequently followed with biennial determinations of (among other things) renal function and albuminuria status over a period of 18 years. For this report we used the last available normoalbuminuric samples from our previous matched case-control/repeated-measures design obtained from a subsample of EDC patients who never developed any evidence of nephropathy (“normals”, N), and patients who subsequently developed microalbuminuria (MA), either intermittent or persistent in repeated assessments of microalbuminuria over a period exceeding 20 years since diagnosis and 18 years under follow up. Urine was thus collected at the end of EDC follow-up in the first group and the two years before the detection of microalbuminuria in the latter group. In the latter group, microalbuminuria was present either on an intermittent or persistent basis in all subsequent visits (up to 18 years) for the EDC cohort. Microalbuminuria was defined as 20–200 μg/min in at least two of three timed urinations (24 h, overnight, and 4 h clinic visit). RNA was isolated from the urine using the miRNeasy kit (Qiagen, Germantown, MD, USA) and microRNA profiles were generated with real-time PCR as previously described [22]. In this analysis we do not differentiate between intermittent and persistent microalbuminuria as our interest lies in the clinical classification of urine samples at only a single point in time. The raw de-identified miRNA data for this analysis are available as supplementary information in our previous publication [22].

### 2.2. Associations between Gender, Microalbuminuria and microRNA Fold Changes

Quantitative Polymerase Reaction (qPCR) quantification cycle values (C_q_) were analyzed with a linear regression model to yield normalized gender-adjusted threshold cycle differences between the N and MA groups for all microRNA species generating measurable signal in >65% of the urine samples. The model used replicates measurements from three microRNAs (miR-423-5p, miR-103, miR-191), three small RNAs (U6, SNORD38B, SNORD38A) and a spike-in control (UniSP3) to supply a panel specific normalization factor that adjusted the difference in threshold crossing value (ΔC_q_) of each microRNA between the two patient groups. ΔC_q_s were converted to relative fold changes (FC) using the delta-delta method [25]: Fold Change = 2^−ΔΔCq^, where ΔΔC_q_ = ΔC_q_ (microRNA) − ΔC_q_ (UniSp3). For all other microRNAs we used logistic regression to analyze the gender adjusted odds ratio (OR) of a given species yielding a signal above the qPCR detection limit between N and MA groups. Though the interpretation of the output of the linear and logistic regression models differs, both yield a numerical summary which can indicate the relative expression of microRNAs between the two patient groups. Due to the exploratory nature of this report, we estimated the parameters of the regression models by adopting a Bayesian probabilistic viewpoint. In the Bayesian framework, one is concerned with the *a posteriori* determination of unobserved quantities e.g., the effects of microalbuminuria or gender on microRNA expression, by combining *a priori* beliefs about the magnitude of these quantities and the evidence provided by the experimental data. In our Bayesian analyses we supplied non-informative prior distributions for the ΔC_q_ and the OR for the linear and logistic regression respectively. These distributions encoded the prior beliefs that for each microRNA (a) the ΔC_q_ could be any number compatible with the dynamic range of our experimental setup (*i.e.*, for 40 qPCR cycles, ΔC_q_ could be between −80 and +80); and (b) the probability of detection could be any number between 0 and 1. The same non-informative priors were adopted for the effects of gender on each microRNA species considered. This Bayesian approach enabled us to rank microRNAs in decreasing order of evidence for differential expression. Therefore, for each microRNA we computed the posterior odds ratio (POR) of the hypothesis that it exhibits directional changes in expression [22,26]. For the interpretation of posterior odds we adopted the following discretization [27]: 1:1–3:1 (not worth more than a bare mention), 3:1–20:1 (positive), 20:1–150:1 (strong), >150:1 (very strong). Bayesian analyses were carried out in WinBUGS/JAGS (code available from the first author).

### 2.3. Target and Pathway Analyses

Target and pathway analyses were used to gain a better understanding of our miRNA biomarker associations. This is an acceptable bioinformatics approach to the identification of cardiovascular miRNA targetsomes [28], which we applied in our previous study [22]. We used the discretized posterior odds ratio to select microRNAs for reporting and to guide functional profiling of microRNA targets predicted by at least 2 of 3 algorithms (miRanda, release August 2010, TargetScan, release 6, and miRDB, version 4.0). To gain a better understanding of our results we used biclustering [29] to group microRNAs according to both the evidence for differential expression and common gene targets they may bind to. The latter, as well as the targets of microRNAs with very strong evidence for differential expression, were mapped to the REACTOME [30] manual ontology of biological pathways and analyzed for enriched terms. Enrichment analysis was carried out with the hypergeometric test using a false discovery rate cutoff of 0.05 to control the number of false positives. R version 3.0.1 and the *Bioconductor* package were used for term enrichment analyses.

### 2.4. Construction of a microRNA Prognostic Index for Microalbuminuria

To construct the microRNA prognostic index, we set the C_q_ value for all microRNAs that failed to yield a detectable signal to 40, *i.e.*, the highest number of qPCR cycles that we run during the experiments. This allowed us to use the entire set of measurements on 722 unique microRNAs as features for the construction of the prognostic index. Subsequently we calculated the linear feature *F* = 40 − C_q_ for each microRNA and each sample in the dataset. Each linear feature is thus related to the amount of the corresponding microRNA species, with higher feature values corresponding to higher urinary concentrations. We used the values of the linear features and their statistical interaction with gender as regressors/predictors in a logistic regression for the outcome of future development of microalbuminuria. Due to the large number of predictors (1445 including the 722 microRNA measurements, gender and the statistical interaction between gender and microRNA species) and the smaller number of measurements (*n* = 27 samples) we used elastic net (EN) regularized regression to select those most predictive of the outcome of interest. The elastic net is a method for automatically selecting features for predictive models that is robust to the presence of high correlations among them [31]. Such group behavior appears plausible for microRNAs with similar binding sequences which belong to the same family, providing the major justification for its use in this work. We used leave-one-out crossvalidation (LOCV) to both fit the EN and assess the predictive performance of the resulting prognostic index. LOCV involves fitting the model to all possible datasets obtained by holding back one data point and then comparing model predictions against the datum that was left out. By this process, the relative contribution of the different features to prediction of the outcome is determined leading to parsimonious prognostic models with high internal validity for the data at hand. For the purposes of this publication we built two prognostic models, one in which the features were equally weighted during EN fitting (“concentration-only model”) and a second one in which features were unequally weighted according to the number of kidney-relevant genes the corresponding microRNAs are predicted to bind to (“concentration-binding model”). Such genes were identified from the Renal Gene Ontology (RGO), a public manually annotated resource of genes implicated in renal disease and development [32]. Previous work has shown that RGO-based annotation may improve [33] the interpretation of gene expression data from kidney biopsies in patients with diabetic nephropathy [34], but to our knowledge this is the first application of the RGO to enhance microRNA analyses. LOCV was used to determine the optimal values of the weights in the concentration-binding model and all models with equal predictive performance were averaged to derive a composite one that is reported herein. All EN analyses were performed in R version 3.0.1 (package glmnet) with Bioconductor packages (UniProt.ws, org.Hs.eg.db, biomaRt) for identifying genes with kidney-relevant RGO annotation terms obtained by querying the UniProt-GOA database [35] using the QuickGo web interface.

### 2.5. Validation of miRNA Features in Type 1 Diabetes

We validated the miRNA features in the identified signatures using two different datasets: a study of 24 age- and hemoglobin-A1C-matched patients with Type 1 diabetes with or without microalbuminuria [20] (available as dataset GSE48318 in the Gene Expression Omnibus, GEO) and a study of patients with hypertensive renal disease and normal controls [23] (GSE28283 and GSE28344). These datasets enabled us to answer the following questions: (a) whether the miRNA changes we describe in our cohort are validated in another cohort of patients with T1 diabetes and (b) whether the predictive performance of miRNAs is more specific for diabetes rather than early non-diabetic kidney disease.

Note that the different techniques used in these two datasets (TaqMan [20] and Affymetrix assays [23]) and different source material (exosomes, kidney biopsy *vs.* total urine) preclude a direct validation of the proposed model in these two external samples. Such a validation would require that one make a large number of unverifiable assumptions to develop a rather complex calibration strategy among measurements obtained with different protocols and measurement systems. Instead, we computed the average performance (Area Under the Receiver Operating Characteristic, AUC-ROC) of miRNAs found in our signatures in these two datasets relative to AUC obtained in the development cohort.

## 3. Results

We assessed 30 patients, *i.e.*, 10 “normals” and 20 who subsequently developed MA, either intermittent (10) or persistent (10), and obtained good quality RNA in all except 3 samples from the MA group. Patients in the normal group had a longer disease duration (median 33.6 *vs.* 22.7) and were older (median age 41.8 *vs.* 24.8 years) relative to participants in the MA group; there was an equal percentage of men and women in the two groups. Characteristics and comorbidities of the individuals who were profiled are shown in Table 1. No patients were receiving an angiotensing converting enzyme inhibitor and only one in the N group was on an angiotensin receptor blocker (patient 21) or an low density lipoprotein (LDL)-lowering agent (patient 23).

**Table 1:** Patient demographics.

ID	Group	Sex	Age	HbA1c	Duration	Cycle	CAD	Stroke	PVD	Neuro	Retino	HTN
1	IMA	F	27.4	10.4	19.2	3	-	-	-	-	-	-
2	PMA	F	22.7	11.8	20.25	3	-	-	-	-	-	-
3	IMA	F	29.9	11.4	18.6	5	-	-	-	-	-	-
4	PMA	F	26.3	13.1	18	5	-	-	-	-	+	-
5	IMA	F	24	10	21.2	2	-	-	-	-	-	-
6	IMA	F	24.3	14.3	19.7	2	-	-	-	-	-	-
7	IMA	F	26.9	10.4	18.5	2	-	-	-	-	-	-
8	PMA	F	25.2	8.2	12.9	2	-	-	-	-	-	-
9	IMA	M	30.66	11	19.97	3	-	-	-	-	+	-
10	PMA	M	23.16	11.5	22.05	3	-	-	+	-	-	-
11	IMA	M	41.7	6.6	30.54	6	-	-	-	-	-	-
12	PMA	M	38.97	5.2	31.54	2	-	-	-	-	+	-
13	IMA	M	39.08	12.4	24.52	6	-	-	-	-	-	-
14	PMA	M	28.35	11.6	27.01	4	-	-	-	-	+	-
15	PMA	M	27.16	13.9	24.3	2	+	-	-	-	+	-
16	IMA	M	23.13	12.1	9.77	2	-	-	-	-	-	-
17	PMA	M	22.8	13	12.9	3	-	-	-	-	-	-
18	N	F	40.32	7.1	29.63	10	-	-	-	+	-	-
19	N	F	48.93	8.3	36.73	10	-	-	-	+	+	-
20	N	F	51.16	8	46.96	10	-	-	+	+	+	-
21	N	F	39.45	7.9	29.77	10	-	-	-	-	-	-
22	N	F	41.19	9.8	38.13	10	+	-	+	+	+	-
23	N	M	48.72	6.6	33.76	10	-	-	-	-	-	-
24	N	M	42.46	9.8	33.4	10	+	-	-	-	-	+
25	N	M	42.5	8.2	36	10	-	-	-	-	-	-
26	N	M	35.35	9.1	28.53	10	+	-	-	+	+	-
27	N	M	38.54	7.7	27.81	10	-	-	-	-	-	-

IMA: intermittent microalbuminuria; PMA: persistent microalbuminuria; N: normal; HbA1c: glycated hemoglobin; PVD: peripheral vascular disease; CAD: coronary artery disease; HTN: hypertension; Neuro: Neuropathy; Retino: Retinopathy; Duration: time since Type 1 diabetes diagnosis; Cycle: number of biennial cycles since enrollment (visit 0: baseline; visit 10: the visit at the 18th year).

**Figure 1:**
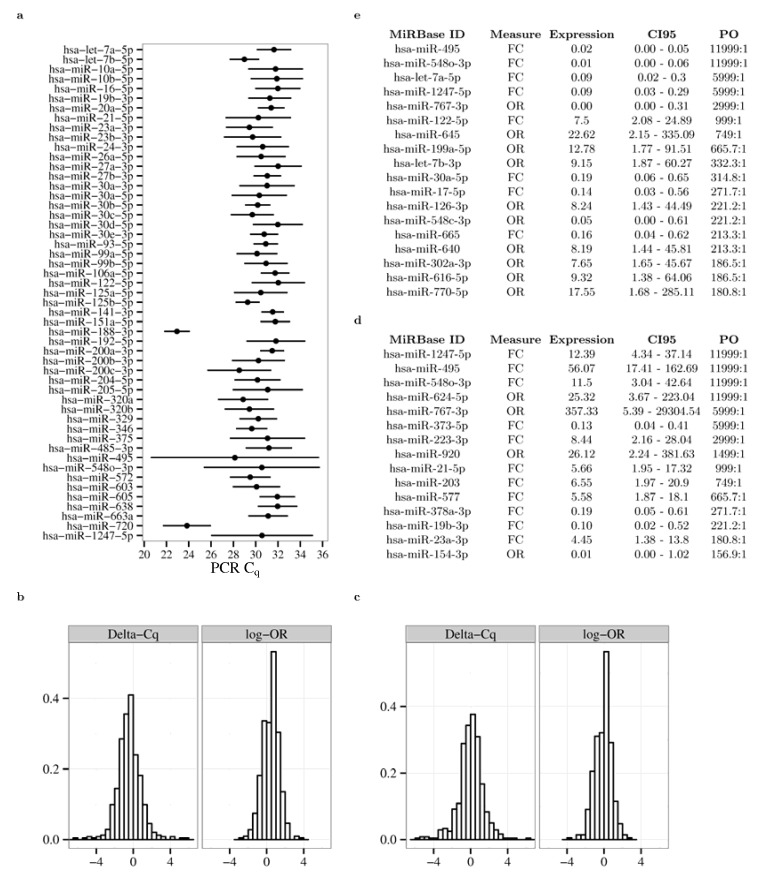
Expression levels of microRNAs in the urine of patients with Type 1 diabetes and associations between subsequent development of microalbuminuria and gender. (**a**) microRNAs with the highest (top 10%) expression (mean ± 1 standard deviation) in the urine of patients who never develop nephropathy; (**b**) histograms of the Delta C_q_ and empirical log odds ratios of detection between the microalbuminuric and normal groups; (**c**) histograms of the Delta Cq and empirical log odds ratios of detection between men and women; (**d**) differences in expression in women *versus* men; (**e**) differences in expression level between MA (microalbuminuric) and N (normal) group. In (**d**,**e**), expression changes (Expression) are given either as fold changes (FC), or odds ratios (OR) of exceeding the detection limit and associated 95% credible interval (CI95). Expression values greater than one and less than one indicate overexpression and underexpression, respectively. PO: posterior odds of the hypothesis that a given microRNA exhibits concentration changes are in the direction indicated by the expression value *vs.* the opposite direction. C_q_: qPCR quantification cycle (threshold crossing) value.

Members of the let-7, miR-10, miR-23, miR-30, miR-200 families were among the microRNAs with the highest expression in the urine of the N-group (Figure 1a). Histograms of the unadjusted (sample) differences in C_q_ and the log-odds (log-OR) of obtaining a measurable signal between the MA and N groups are shown in Figure 1b. Figure 1c shows the same data for the comparison between men and women. We found very strong evidence for differential expression (PO > 150:1) for 18 microRNAs in the MA *vs.* the N-group comparison Figure 1e and for 15 microRNAs in women, as shown in Figure 1d (the analyses for all 722 microRNAs assessed are given in the Supplementary Tables). Four microRNAs (miR-1247-5p, miR-495, miR-548o-3p and miR-767-3p) exhibited reciprocal changes in expression in the MA (decreased) *vs.* the gender (increased) comparisons.

**Figure 2:**
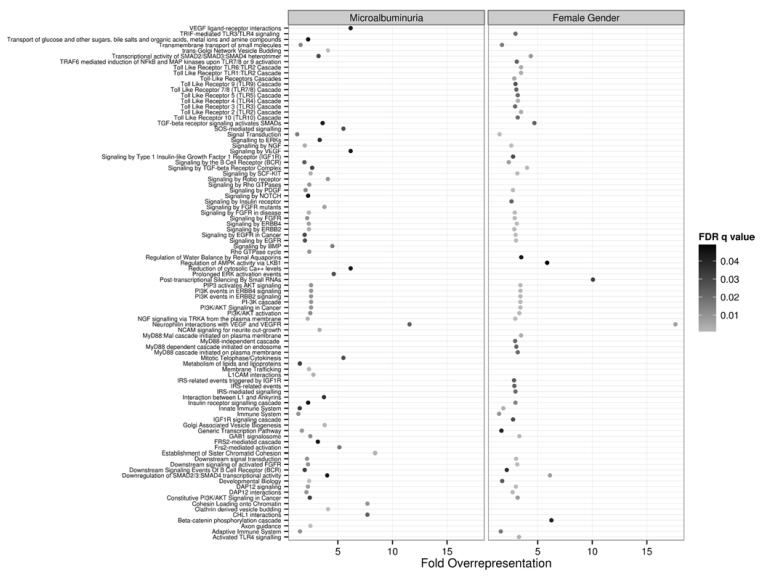
Over-representation analysis in the REACTOME ontology of putative targets of differentially expressed microRNAs in patients with MA and women plotted side by side. The fold-overrepresentation of terms in each pathway is plotted in the x-axis, while the statistical significance (False Discovery Rate adjusted *q* value) is encoded in gray scale.

Term enrichment analysis highlighted a number of distinct pathways involving growth factor signaling (VEGF, EGF, FGF, TGF, NGF, SMAD), insulin/IGF and PI3K signaling, apoptosis, innate immunity (Toll/TLR cascade), transmembrane transport and other pathways as potential targets of these differentially expressed microRNAs (Figure 2). Although the majority of these pathways were over-represented in both comparisons, they were some exceptions e.g., VEGF (only in MA) and TLR 2–10 signaling (women).

**Figure 3:**
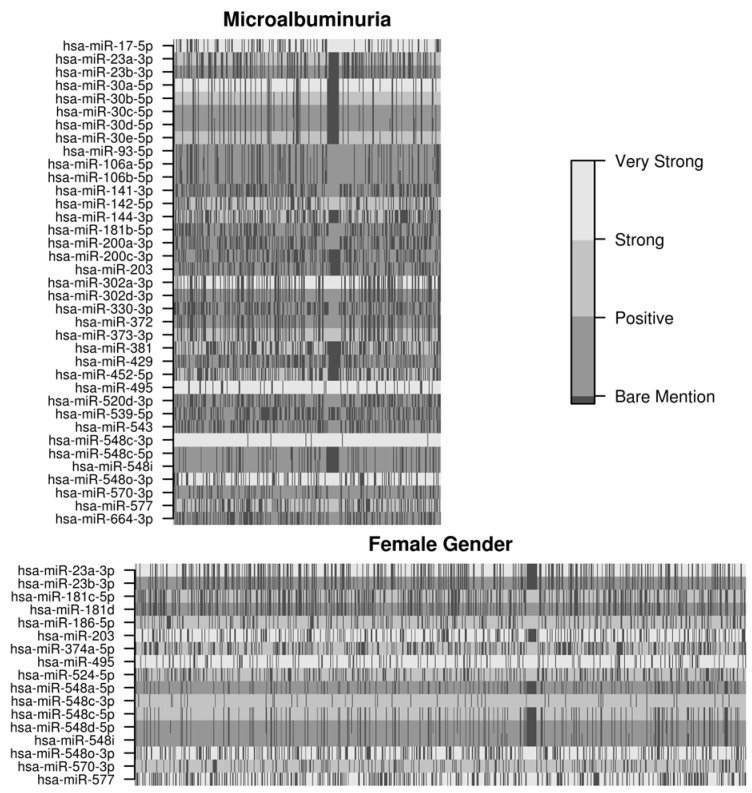
Simultaneous clustering (biclustering) of the evidence for differential expression microRNAs and their predicted targets. Biclusters are plotted in gray scale according to the evidence of differential regulation (posterior odds); for microRNAs not predicted to bind to a given target, the lowest possible evidence for differential regulation was assigned.

Biclustering of the evidence for differential expression and predicted gene targets for all 722 microRNAs and 17,938 targets suggested a single bicluster for MA status and female gender (Figure 3) consisting of 37 microRNAs by 360 targets and 17 microRNAs by 878 targets, respectively. These two biclusters shared only a few microRNAs (miR-23a/b, miR-495, miR-548c-3p/5p, miR-548o-3p, miR-570-3p, miR-577).

**Figure 4:**
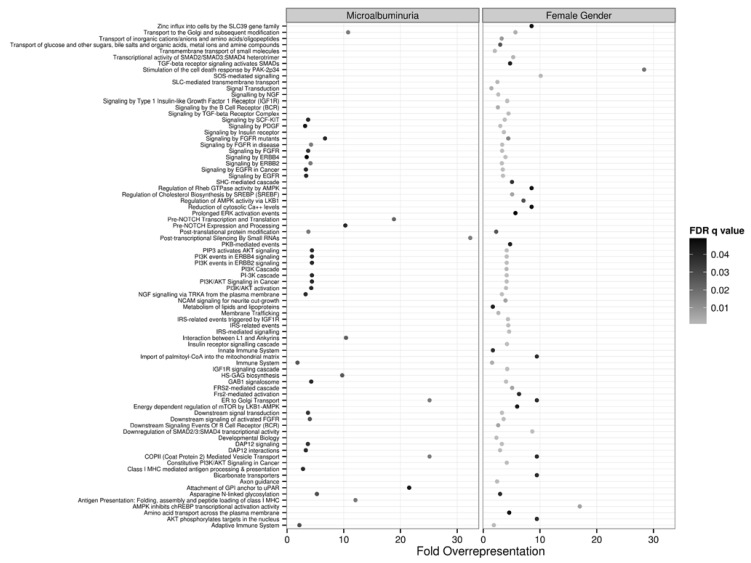
Over-representation analysis in the REACTOME ontology of putative targets of microRNAs in the biclusters identified in the comparisons between microalbuminuria (MA) *vs.* Normal (N) groups and women *vs.* men plotted side by side. The fold-overrepresentation of terms in each pathway is plotted in the x-axis, while the statistical significance (False Discovery Rate-adjusted *q* value) is encoded in gray scale.

Enrichment analysis of the bicluster targets (Figure 4) highlighted the majority of the pathways in Figure 2. Of note, the most enriched pathway (>30 fold) in patients with MA was that of post-transcriptional silencing by small RNAs, while TGF and SMAD signaling appeared to be over-represented in the bicluster of females.

Notwithstanding these associations, only a small number of microRNAs were sufficient to predict the future development of microalbuminuria. In particular, a microRNA signature of 11 microRNAs (Table 1, concentration-only model) exhibited a high degree of predictive accuracy (cross-validated misclassification rate of 11%). MicroRNAs in this signature were among the most highly expressed species in the urine (miR-30b-3p, 122-5p, 192-5p, 200b-5p, miR-495, 548-3p, 720), had high evidence for differential expression in normoalbuminuric samples (miR-495, 548o-3p, 122-5p, 126-3p) or belonged to the same bicluster for gender (miR-30b-50, 495, 548o-3p).

The median (Interquartile Range) number of genes belonging to the Gene Ontology and its renal subset targeted by the 722 microRNAs considered in this analysis were 1169 (1295.25) and 54 (63) respectively. Weighing the microRNA measurements according to the number of their predicted renal gene targets resulted in a larger microRNA signature of 13 species (Table 1, concentration-binding model) with slightly higher prognostic accuracy (cross-validated misclassification rate of 7.4%) for the future development of microalbuminuria. The two signatures exhibited a large degree of overlap with nine common microRNAs and similar quantitative contribution (sign and magnitude of log-odds) for the classification of patient samples. Both signatures included a small number of gender-specific microRNA contributions.

We considered the following miRNAs for validation: those whose expression differed significantly in adjusted analyses (Figure 1e, signature D) and the two signatures (C: concentration only, CB: Concentration-Binding miRNAs) in Table 2. The average AUC (range) for the miRNAs in the development cohort was 0.643 (0.622–0.806), 0.721 (0.559–0.847) and 0.692 (0.559–0.847) for the D, C and CB respectively. Not all miRNAs identified in our analyses were profiled in the validation cohorts since the relevant probes were not included in the corresponding qPCR and microarray platforms. miRNAs not profiled included hsa-miR-548o-3p, hsa-miR-767-3p, hsa-miR-1247-5p, hsa-miR-645, hsa-let-7b-3p, hsa-miR-30a-5p, hsa-miR-665, hsa-miR-640, hsa-miR-616-5p and hsa-miR-770-5p from the signature D and hsa-miR-105-3p, hsa-miR-122-3p, hsa-miR-1972 from signatures C and CB. In the microalbuminuric development cohort the average (range) of individual AUCs were 0.786 (0.50–1.0), 0.714 (0.5–1.0) and 0.714 (0.5–1.0) for the D, C and CB signatures. In the hypertensive validation cohort the corresponding figures were: 0.667 (0.533–0.817), 0.676 (0.533–0.767) and 0.679 (0.533–0.767) respectively for the D, C and CB signatures.

**Table 2:** Predictive models for the future development of microalbuminuria.

Feature	Log-Odds ^ǂ^	
	Concentration—Only Model	Concentration—Binding Model
Intercept	2.725	3.313
hsa-miR-105-3p	−0.125	−0.196
hsa-miR-122-3p		0.022
hsa-miR-124-3p		0.003
hsa-miR-126-3p		0.045
hsa-miR-1972	−0.003	−0.054
hsa-miR-28-5p	−0.316	−0.682
hsa-miR-30b-5p	−0.008	
hsa-miR-363-3p	−0.141	−0.009
hsa-miR-424-5p	−0.069	
hsa-miR-486-5p	0.083	0.212
hsa-miR-495	−0.045	−0.028
hsa-miR-548o-3p	−0.055	
hsa-miR-122-5p X Women		0.007
hsa-miR-192-5p X Women	0.033	0.03
hsa-miR-200c-3p X Women		0.07
hsa-miR-548o-3p X Women	−0.296	−0.498
hsa-miR-720 X Women	0.059	0.018

^ǂ^ Log-Odds ratios are coefficients that multiply the features (40-C_q_) for each of the microRNAs measured in the urine. These terms are then added together to give an overall log-odds score which when exponentiated yields the odds of microalbuminuria development for a given sample. These microRNA measurements carry a different prognostic implication for women. For these microRNAs the log-odds multiply the corresponding feature only for women.

## 4. Discussion

In this study we report differences in the microRNA profiles in Type 1 diabetes patients without nephropathy and patients who subsequently develop microalbuminuria. These differences are apparent years before those patients manifest microalbuminuria, suggesting that microRNA signatures may serve as a useful and accurate prognostic marker for the development of incipient diabetic nephropathy or endothelial dysfunction. Furthermore, there appear to exist differences in the microRNA profiles between male and female patients, which to our knowledge is a novel observation in diabetes. Targets of these differentially co-expressed microRNAs map to biological pathways of known relevance to the development of diabetic kidney disease, providing a *post hoc* context for the interpretation of the reported changes. miRNAs identified by our analyses maintained their predictive performance and appear to be relatively specific for the microalbuminuria of Type 1 diabetes when validated in two cohorts of diabetic and hypertensive patients, respectively.

The urine of diabetic patients without nephropathy appears to have detectable levels of microRNAs of known relevance to renal morphology, physiology and pathophysiology [15,16,17]. More specifically associations have been previously reported with kidney development (miR-200, miR-30a, miR-17-5p, let-7a, miR-23a, miR-26a, miR-24), regulation of water transport (miR-320a), osmoregulation (miR-200b) and sodium/potassium transport (miR-192). Other microRNAs have been implicated in disease phenotypes including hypertension (miR-200a/b, miR-141, miR-192, miR-205), renal fibrosis (miR-192, miR-200a/b, miR-21), polycystic kidney disease (miR-17), renal cancer (miR-17-5p, miR-122-5p) and even diabetic nephropathy (miR-192, miR-21). Some of these microRNAs (miR-21, miR-29c, miR-30d, miR-124a, miR-320, miR-375) have been shown to be glucose-induced in non-renal contexts (pancreas, adipocytes, endothelial cells) or to modulate insulin sensitivity or lipoprotein metabolism (miR-122). Thus, it appears that in the absence of diabetic kidney disease, the urinary microRNome harbors a number of microRNAs that are contextually related to renal pathophysiology and the diabetic milieu. An interesting hypothesis that deserves further study is that these microRNAs underlie the apparent resistance of some patients to the development of renal disease despite the long duration of diabetes.

These observations suggest that perturbations from the normal urinary microRNome may open a non-invasive window into these processes, even before microalbuminuria becomes manifest. In that regard, our data provide support for differences in microRNA expression at least two years before the detection of microalbuminuria for the first time. Although many of the differentially expressed microRNAs (e.g., miR-126, miR-141-3p, miR-429, miR-373-3p) have been implicated in renal disease and hypertension [15,16,17,22], the renal or diabetes context of others (notably miR-495, miR-1247, miR-548o) is currently unexplored in the literature. A recent paper reported differences in urinary exosomal microRNAs [20] between normoalbuminuric and microalbuminuric Type 1 diabetes patients with incipient nephropathy. Similar to our report [22], this work studied patients with long duration of disease and found evidence for over-expression for miR-130a(-3p) and miR-145(-5p), while miR-155(-5p) and miR-424(-5p) were under-expressed. We found positive (POR of 13.6:1) and strong (POR 126:1) for over-expression of miR-145-5p and under-expression of miR-424-5p (Table S1) but minimal support for differential expression of miR-130a-3p and miR-155-5p. The lack of complete concordance between the findings of these studies is not surprising, though, given the different microRNA sources (exosomes *vs.* total urine), gender (all men in [20], an equal proportion of men and women in our report) and age (late 50s in [20] and early 40s in our work). Notwithstanding these observations, the miRNAs identified in our analyses are validated against this cohort.

We should note that the inter-individual changes in microRNA expression only partially overlap with our previous report of intra-individual associations during the progression from normoalbuminuria to intermittent or persistent microalbuminuria [22]. This pattern suggests that different microRNAs are involved in determining inter-individual susceptibility to the development of microalbuminuria and its onset among those susceptible. In the absence of experimental work that would provide a context for the interpretation of differential microRNA expression, it is re-assuring to note that the predicted targets of differentially expressed microRNAs map to pathways that are implicated in the development and progression of diabetic kidney disease (DKD) and renal fibrosis: the growth factor TGF/FGF/PDGF/VEGF, SMAD signaling, and PI3K/AKT signaling. Hence the expression changes reported here appear to be compatible with our current understanding of DKD, and consistent with recently emerging themes [36] in the field (e.g., the role of inflammation, innate immunity and toll receptor signaling).

In this work we describe gender-related associations in the urine of patients with Type 1 diabetes. Although gender-associated changes have been described in patients with the metabolic syndrome [37] and estrogen dependent microRNA regulation is well established [38] we are not aware of any previous reports in diabetes or microalbuminuria. With a few exceptions (e.g., miR-19b, miR-21-5p, miR-223-3p, miR-378 [39]), many of the microRNAs identified as having a gender-related expression level have not been adequately studied so as to provide an adequate context for the interpretation of such changes. Thus, it is interesting to note that the predicted targets of these microRNAs largely map to the same pathways as the microRNAs associated with subsequent development of microalbuminuria. Whether this observation relates to the weakening or even loss of the protective effect of female gender on the progression of DKD [40], seen in almost all primary renal diseases [41,42,43], cannot be established from this work and requires further study.

Early treatment of patients with Type 1 diabetes with inhibitors of the renin angiotensin aldosterone system is thought to confer some advantage in delaying the onset of diabetic renal complications in those with microalbuminuria but not those with normoalbuminuria [44]. Hence, non-albumin biomarkers that identify patients prior to the development microalbuminuria may offer a unique approach towards individualizing treatment by targeting renin-angiotensin-aldosterone blockers to those who are progressing towards microalbuminuria. However, this is a hypothesis that should be tested in prospective clinical trials before being adopted as a therapeutic strategy in the clinic.

In this work we used urinary measurements of miRNAs, their binding patterns, the recently described Renal Gene Ontology annotation [32] and longitudinal follow-up to develop miRNA biomarkers with some specificity for the microalbuminuric phenotype of Type 1 diabetes. Of note, only a small number of the differentially expressed microRNAs between non-progressing normoalbuminuric patients and those who progressed to microalbuminuria were identified as prognostically significant. This apparent discrepancy is explained by considering the qualifications of a predictive marker: it should not only be expressed at different levels between patient groups but its expression should also have a small inter-individual variability and thus measurement noise. On the other hand, including all miRNAs with the same directional expression changes in the signature is a wasteful strategy. Techniques such as the elastic net take into account the correlations between individual biomarkers and allow the development of more parsimonious composite signatures. In particular, the biomarkers derived by the elastic net are composed of miRNAs that represent two different quantitative behaviours: those that are highly expressed in the urine and those that exhibit large differential changes between the two patient groups. The resulting miRNA signatures were thus shorter (fewer miRNAs) and had a marginally higher predictive performance in the diabetic validation dataset compared to the signature obtained by miRNAs with the highest directional changes. Consideration of target binding patterns in the elastic net algorithm identified miRNAs with the same average AUC in the hypertensive kidney disease validation cohort.

A number of limitations have to be kept in mind in interpreting the results of this study. First, this is a cross-sectional secondary analysis of a previous matched case-control study which explored microRNA changes between stages of diabetic nephropathy. As matching was not preserved in this report, some patient characteristics (e.g., age and duration of disease at urine sampling) differed between patient groups and their effects are confounded in our analysis. Nevertheless, none of the miRNAs identified in a study of older *vs.* younger individuals [45] (e.g., miR-151a-3p, 1248 or 181a-5p) exhibited significant expression changes in our study. To the extent that the aging renal phenotype shares common pathways with diabetic nephropathy [46,47], one should expect our study to have decreased sensitivity in terms of identifying microRNA associated with DKD. Second, the relative small number of patients makes the precise quantification of changes in expression rather challenging, which is reflected in the large credible intervals for some of the microRNAs and their apparent lack of prognostic significance. For these microRNAs the present study should be regarded as providing evidence about the general direction in the change expression (up or down), not its actual magnitude. Third, our patients have never had renal biopsies that would allow us to correlate the urine microRNome with specific tissue signatures or even determine their cellular source. In the absence of such data we had to heavily rely on bioinformatic tools to provide a *post hoc* context for the interpretation of expression changes. Fourth, our study used microalbuminuria as evidence for renal involvement in the patients we examined [48]. Nevertheless, microalbuminuria is also a risk factor for cardiovascular disease in patients with or without diabetes and currently there exist no tests that can differentiate between these two potential causes [49]. Hence, the associations and prognostic models we develop may not be specific for diabetic renal disease, but rather identify patients with microalbuminuria-manifesting endothelial dysfunction [50]. However, only one patient in our study had hypertension, while individual miRNA signatures had a higher predictive performance in the validation dataset of diabetes compared to that of hypertensive kidney disease. Future studies should examine differential miRNA expression in cohorts of patients with diabetes (with or without hypertension) and hypertensive non-diabetic kidney disease to further characterize the specificity of miRNAs for different microalbuminuric clinical phenotypes.

Due to these limitations, our findings should be regarded principally as hypothesis generating. Nevertheless, subsequent investigations may consider additional background information and ultimately corroborate findings more weakly supported by our data or even refute others that appear to be strongly associated. In particular, validation of our microRNA signature prognostic models against clinical outcomes of either cardiovascular or progressive renal disease may provide a novel way to differentiate between the sources of micro-albuminuria or even target therapies in patients with Type 1 diabetes.

## 5. Conclusions

In summary, we characterized the urinary microRNome in normoalbuminuric patients with Type 1 diabetes and found specific microRNAs mapping to pathways of known pathophysiological significance for DKD, to be differentially expressed. As both intermittent and persistent MA carry a heightened risk for disease progression [51,52] early identification of patients likely to have defined nephropathy may allow for earlier institution of renoprotective therapies [44]. Further longitudinal studies are needed to clarify the potential utility of urinary microRNome in the early diagnosis of nephropathy and treatment selection or monitoring.

## Supplementary Files

Supplementary File 1

Supplementary File 1

## References

[1] Gross J.L., de Azevedo M.J., Silveiro S.P., Canani L.H., Caramori M.L., Zelmanovitz T. (2005). Diabetic Nephropathy: Diagnosis, Prevention, and Treatment. Diabetes Care.

[2] Sacks D.B., Arnold M., Bakris G.L., Bruns D.E., Horvath A.R., Kirkman M.S., Lernmark A., Metzger B.E., Nathan D.M. (2011). Guidelines and Recommendations for Laboratory Analysis in the Diagnosis and Management of Diabetes Mellitus. Clin. Chem..

[3] Fioretto P., Mauer M. (2007). Histopathology of diabetic nephropathy. Semin. Nephrol..

[4] Bader R., Bader H., Grund K.E., Mackensen-Haen S., Christ H., Bohle A. (1980). Structure and function of the kidney in diabetic glomerulosclerosis. Correlations between morphological and functional parameters. Pathol. Res. Pract..

[5] Caramori M.L., Kim Y., Huang C., Fish A.J., Rich S.S., Miller M.E., Russell G., Mauer M. (2002). Cellular Basis of Diabetic Nephropathy 1. Study Design and Renal Structural-Functional Relationships in Patients with Long-Standing Type 1. Diabetes.

[6] Najafian B., Kim Y., Crosson J.T., Mauer M. (2003). Atubular Glomeruli and Glomerulotubular Junction Abnormalities in Diabetic Nephropathy. J. Am. Soc. Nephrol..

[7] Najafian B., Crosson J.T., Kim Y., Mauer M. (2006). Glomerulotubular Junction Abnormalities are Associated with Proteinuria in Type 1 Diabetes. J. Am. Soc. Nephrol..

[8] Gonzalez Suarez M.L., Thomas D.B., Barisoni L., Fornoni A. (2013). Diabetic nephropathy: Is it time yet for routine kidney biopsy?. World J. Diabetes.

[9] Alter M.L., Kretschmer A., von Websky K., Tsuprykov O., Reichetzeder C., Simon A., Stasch J.-P., Hocher B. (2012). Early urinary and plasma biomarkers for experimental diabetic nephropathy. Clin. Lab..

[10] Astor B.C., Matsushita K., Gansevoort R.T., van der Velde M., Woodward M., Levey A.S., de Jong P.E., Coresh J., el-Nahas M., Eckardt K.-U. (2011). Lower estimated glomerular filtration rate and higher albuminuria are associated with mortality and end-stage renal disease. A collaborative meta-analysis of kidney disease population cohorts. Kidney Int..

[11] Gansevoort R.T., Matsushita K., van der Velde M., Astor B.C., Woodward M., Levey A.S., de Jong P.E., Coresh J., el-Nahas M., Eckardt K.-U. (2011). Lower estimated GFR and higher albuminuria are associated with adverse kidney outcomes. A collaborative meta-analysis of general and high-risk population cohorts. Kidney Int..

[12] Matsushita K., van der Velde M., Astor B.C., Woodward M., Levey A.S., de Jong P.E., Coresh J., Gansevoort R.T. (2010). Association of estimated glomerular filtration rate and albuminuria with all-cause and cardiovascular mortality: A collaborative meta-analysis of general population cohorts. Lancet.

[13] Van Roosbroeck K., Pollet J., Calin G.A. (2013). miRNAs and long noncoding RNAs as biomarkers in human diseases. Expert Rev. Mol. Diagn..

[14] Mraz M., Malinova K., Mayer J., Pospisilova S. (2009). MicroRNA isolation and stability in stored RNA samples. Biochem. Biophys. Res. Commun..

[15] Khella H.W.Z., Bakhet M., Lichner Z., Romaschin A.D., Jewett M.A.S., Yousef G.M. (2013). MicroRNAs in kidney disease: An emerging understanding. Am. J. Kidney Dis..

[16] Schena F.P., Serino G., Sallustio F. (2014). MicroRNAs in kidney diseases: New promising biomarkers for diagnosis and monitoring. Nephrol. Dial. Transplant..

[17] Chandrasekaran K., Karolina D.S., Sepramaniam S., Armugam A., Wintour E.M., Bertram J.F., Jeyaseelan K. (2012). Role of microRNAs in kidney homeostasis and disease. Kidney Int..

[18] Alvarez M.L., Distefano J.K. (2013). The role of non-coding RNAs in diabetic nephropathy: Potential applications as biomarkers for disease development and progression. Diabetes Res. Clin. Pract..

[19] Yang Y., Xiao L., Li J., Kanwar Y.S., Liu F., Sun L. (2013). Urine miRNAs: Potential biomarkers for monitoring progression of early stages of diabetic nephropathy. Med. Hypotheses.

[20] Barutta F., Tricarico M., Corbelli A., Annaratone L., Pinach S., Grimaldi S., Bruno G., Cimino D., Taverna D., Deregibus M.C. (2013). Urinary Exosomal MicroRNAs in Incipient Diabetic Nephropathy. PLoS ONE.

[21] Osipova J., Fischer D.-C., Dangwal S., Volkmann I., Widera C., Schwarz K., Lorenzen J.M., Schreiver C., Jacoby U., Heimhalt M. (2014). Diabetes-associated microRNAs in paediatric patients with Type 1 diabetes mellitus: A cross-sectional cohort study. J. Clin. Endocrinol. Metab..

[22] Argyropoulos C., Wang K., McClarty S., Huang D., Bernardo J., Ellis D., Orchard T., Galas D., Johnson J. (2013). Urinary microRNA profiling in the nephropathy of Type 1 diabetes. PLoS ONE.

[23] Marques F.Z., Campain A.E., Tomaszewski M., Zukowska-Szczechowska E., Yang Y.H.J., Charchar F.J., Morris B.J. (2011). Gene Expression Profiling Reveals Renin mRNA Overexpression in Human Hypertensive Kidneys and a Role for MicroRNAs. Hypertension.

[24] Miller R.G., Secrest A.M., Ellis D., Becker D.J., Orchard T.J. (2013). Changing Impact of Modifiable Risk Factors on the Incidence of Major Outcomes of Type 1 Diabetes: The Pittsburgh Epidemiology of Diabetes Complications Study. Diabetes Care.

[25] Pfaffl M.W. (2001). A new mathematical model for relative quantification in real-time RT-PCR. Nucleic Acids Res..

[26] Argyropoulos C., Nikiforidis G.C., Theodoropoulou M., Adamopoulos P., Boubali S., Georgakopoulos T.N., Paliogianni F., Papavassiliou A.G., Mouzaki A. (2004). Mining microarray data to identify transcription factors expressed in naïve resting but not activated T lymphocytes. Genes Immun..

[27] Kass R.E., Raftery A.E. (1995). Bayes Factors. J. Am. Stat. Assoc..

[28] Fiedler J., Gupta S.K., Thum T. (2011). Identification of cardiovascular microRNA targetomes. J. Mol. Cell. Cardiol..

[29] Turner H., Bailey T., Krzanowski W. (2005). Improved biclustering of microarray data demonstrated through systematic performance tests. Comput. Stat. Data Anal..

[30] Matthews L., Gopinath G., Gillespie M., Caudy M., Croft D., de Bono B., Garapati P., Hemish J., Hermjakob H., Jassal B. (2009). REACTOME knowledgebase of human biological pathways and processes. Nucleic Acids Res..

[31] Zou H., Hastie T. (2005). Regularization and variable selection via the elastic net. J. R. Stat. Soc. Ser. B Stat. Methodol..

[32] Alam-Faruque Y., Dimmer E.C., Huntley R.P., O’Donovan C., Scambler P., Apweiler R. (2010). The renal gene ontology annotation initiative. Organogenesis.

[33] Alam-Faruque Y., Hill D.P., Dimmer E.C., Harris M.A., Foulger R.E., Tweedie S., Attrill H., Howe D.G., Thomas S.R., Davidson D. (2014). Representing Kidney Development Using the Gene Ontology. PLoS ONE.

[34] Baelde H.J., Eikmans M., Doran P.P., Lappin D.W.P., de Heer E., Bruijn J.A. (2004). Gene expression profiling in glomeruli from human kidneys with diabetic nephropathy. Am. J. Kidney Dis..

[35] Barrell D., Dimmer E., Huntley R.P., Binns D., O’Donovan C., Apweiler R. (2009). The GOA database in 2009—An integrated Gene Ontology Annotation resource. Nucleic Acids Res..

[36] Wada J., Makino H. (2013). Inflammation and the pathogenesis of diabetic nephropathy. Clin. Sci..

[37] Wang Y.-T., Tsai P.-C., Liao Y.-C., Hsu C.-Y., Juo S.-H.H. (2013). Circulating microRNAs have a sex-specific association with metabolic syndrome. J. Biomed. Sci..

[38] Klinge C.M. (2009). Estrogen Regulation of MicroRNA Expression. Curr. Genomics.

[39] Ganesan J., Ramanujam D., Sassi Y., Ahles A., Jentzsch C., Werfel S., Leierseder S., Loyer X., Giacca M., Zentilin L. (2013). MiR-378 controls cardiac hypertrophy by combined repression of mitogen-activated protein kinase pathway factors. Circulation.

[40] Costacou T., Fried L., Ellis D., Orchard T.J. (2011). Sex Differences in the Development of Kidney Disease in Individuals with Type 1 Diabetes Mellitus: A Contemporary Analysis. Am. J. Kidney Dis..

[41] Neugarten J., Acharya A., Silbiger S.R. (2000). Effect of gender on the progression of nondiabetic renal disease: A meta-analysis. J. Am. Soc. Nephrol..

[42] Neugarten J., Golestaneh L. (2013). Gender and the prevalence and progression of renal disease. Adv. Chronic Kidney Dis..

[43] Cattran D.C., Reich H.N., Beanlands H.J., Miller J.A., Scholey J.W., Troyanov S., Genes, Gender and Glomerulonephritis Group (2008). The impact of sex in primary glomerulonephritis. Nephrol. Dial. Transplant..

[44] Hirst J.A., Taylor K.S., Stevens R.J., Blacklock C.L., Roberts N.W., Pugh C.W., Farmer A.J. (2012). The impact of renin-angiotensin-aldosterone system inhibitors on Type 1 and Type 2 diabetic patients with and without early diabetic nephropathy. Kidney Int..

[45] Hooten N.N., Fitzpatrick M., Wood W.H., De S., Ejiogu N., Zhang Y., Mattison J.A., Becker K.G., Zonderman A.B., Evans M.K. (2013). Age-related changes in microRNA levels in serum. Aging.

[46] Verzola D., Gandolfo M.T., Gaetani G., Ferraris A., Mangerini R., Ferrario F., Villaggio B., Gianiorio F., Tosetti F., Weiss U. (2008). Accelerated senescence in the kidneys of patients with type 2 diabetic nephropathy. Am. J. Physiol. Ren. Physiol..

[47] Wu J., Zhang R., Torreggiani M., Ting A., Xiong H., Striker G.E., Vlassara H., Zheng F. (2010). Induction of diabetes in aged C57B6 mice results in severe nephropathy: An association with oxidative stress, endoplasmic reticulum stress, and inflammation. Am. J. Pathol..

[48] Roscioni S.S., Lambers Heerspink H.J., de Zeeuw D. (2014). Microalbuminuria: Target for renoprotective therapy PRO. Kidney Int..

[49] MacIsaac R.J., Ekinci E.I., Jerums G. (2014). Progressive diabetic nephropathy. How useful is microalbuminuria?: Contra′. Kidney Int..

[50] Glassock R.J. (2010). Is the presence of microalbuminuria a relevant marker of kidney disease?. Curr. Hypertens. Rep..

[51] Galler A., Haberland H., Näke A., Hofer S., Holder M., Raile K., Holl R.W. (2012). Natural course of untreated microalbuminuria in children and adolescents with Type 1 diabetes and the importance of diabetes duration and immigrant status: Longitudinal analysis from the prospective nationwide German and Austrian diabetes survey DPV. Eur. J. Endocrinol..

[52] Amin R., Widmer B., Prevost A.T., Schwarze P., Cooper J., Edge J., Marcovecchio L., Neil A., Dalton R.N., Dunger D.B. (2008). Risk of microalbuminuria and progression to macroalbuminuria in a cohort with childhood onset Type 1 diabetes: Prospective observational study. BMJ.

