# Supplementary material for: Urinary MicroRNA Profiling Predicts the Development of Microalbuminuria in Patients with Type 1 Diabetes
Source: J Clin Med. 2015 Jul 17;4(7):1498–517. doi: 10.3390/jcm4071498 (PMC4519802; doi:10.3390/jcm4071498)
Supplement: Supplementary File 1 [file jcm-04-01498-s001.pdf]

Supplementary Table S1

MiRBase_ID	Effect	Size	CI 95	POR
hsa-miR-495	FC	0.02	0–0.05	11,999:1
hsa-miR-548o-3p	FC	0.01	0–0.06	11,999:1
hsa-let-7a-5p	FC	0.09	0.02–0.3	5999:01:00
hsa-miR-1247-5p	FC	0.09	0.03–0.29	5999:01:00
hsa-miR-767-3p	OR	0	0–0.31	2999:01:00
hsa-miR-122-5p	FC	7.5	2.08–24.89	999:01:00
hsa-miR-645	OR	22.62	2.15–335.09	749:01:00
hsa-miR-199a-5p	OR	12.78	1.77–91.51	665.7:1
hsa-let-7b-3p	OR	9.15	1.87–60.27	332.3:1
hsa-miR-30a-5p	FC	0.19	0.06–0.65	314.8:1
hsa-miR-17-5p	FC	0.14	0.03–0.56	271.7:1
hsa-miR-126-3p	OR	8.24	1.43–44.49	221.2:1
hsa-miR-548c-3p	OR	0.05	0–0.61	221.2:1
hsa-miR-665	FC	0.16	0.04–0.62	213.3:1
hsa-miR-640	OR	8.19	1.44–45.81	213.3:1
hsa-miR-302a-3p	OR	7.65	1.65–45.67	186.5:1
hsa-miR-616-5p	OR	9.32	1.38–64.06	186.5:1
hsa-miR-770-5p	OR	17.55	1.68–285.11	180.8:1
hsa-miR-424-5p	FC	0.19	0.05–0.79	126.7:1
hsa-miR-617	OR	8.8	1.49–82.09	124:01:00
hsa-miR-30e-5p	FC	0.16	0.03–0.65	121.4:1
hsa-miR-23a-3p	FC	0.24	0.07–0.81	95.8:1
hsa-miR-603	FC	0.23	0.07–0.71	95.8:1
hsa-let-7c	FC	0.23	0.07–0.82	92.8:1
hsa-miR-1908	OR	0.07	0–1.02	84.7:1
hsa-miR-124-3p	FC	4.97	1.39–21.07	84.7:1
hsa-miR-411-5p	OR	5.94	1.27–35.25	83.5:1
hsa-miR-92b-5p	OR	5.8	1.19–31.27	72.2:1
hsa-miR-126-5p	OR	5.82	1.27–35.57	71.3:1
hsa-miR-221-5p	OR	5.8	1–32.2	57.8:1
hsa-miR-142-5p	OR	6.24	1.05–37.9	56.7:1
hsa-miR-30b-5p	FC	0.25	0.07–0.93	49.8:1
hsa-miR-329	FC	0.28	0.08–0.9	49.4:1
hsa-miR-577	FC	0.29	0.09–0.94	49.4:1
hsa-miR-381	OR	5.44	0.98–30.06	49.4:1
hsa-miR-302c-5p	OR	6.99	0.89–51.8	45.9:1
hsa-miR-483-3p	OR	5.44	1.02–31.7	45.5:1
hsa-miR-548b-3p	OR	0.12	0.01–1.18	44.1:1
hsa-miR-885-3p	FC	3.21	1.04–9.96	43.8:1
hsa-miR-127-3p	OR	0.02	0–2.13	43.4:1
hsa-miR-567	OR	0.02	0–1.61	41.9:1
hsa-miR-122-3p	OR	5.71	1.02–43.98	38.5:1
hsa-miR-572	FC	0.31	0.1–1.02	37.5:1
hsa-miR-2110	FC	0.33	0.1–1.01	34.9:1
hsa-let-7b-5p	FC	0.31	0.09–1.01	33.1:1
hsa-miR-379-3p	OR	7.82	0.77–125.09	27.6:1
hsa-miR-302d-5p	OR	4.34	0.87–22.87	26.1:1

CI 95, 95% confidence intervals; POR, posterior odds ratio; FC, fold change; OR, odd ratio.

hsa-miR-210	FC	0.26	0.06–1.14	25.7:1
hsa-miR-34a-3p	OR	0.03	0–2.06	25.7:1
hsa-miR-373-3p	OR	6.67	0.72–102.06	24.3:1
hsa-miR-523-3p	OR	4.29	0.83–25.24	23.5:1
hsa-miR-144-3p	OR	7.71	0.68–115.87	22.8:1
hsa-miR-518f-3p	OR	0.04	0–2.68	22.6:1
hsa-miR-96-5p	OR	0.03	0–2.16	22.4:1
hsa-miR-92a-3p	FC	2.89	0.88–9.7	22.3:1
hsa-miR-452-5p	FC	0.28	0.07–1.16	22.3:1
hsa-miR-548m	OR	6.72	0.68–101.05	22.1:1
hsa-miR-328	OR	3.99	0.84–22.71	22:01
hsa-miR-320a	FC	0.36	0.12–1.19	21.7:1
hsa-miR-193a-3p	OR	0.04	0–2.57	21.2:1
hsa-miR-520a-5p	OR	0.04	0–1.87	21:01
hsa-miR-1181	OR	3.79	0.72–17.15	20:01
hsa-miR-132-5p	OR	4.06	0.76–21.91	19.9:1
hsa-miR-149-3p	FC	3.19	0.75–11.28	19.6:1
hsa-miR-345-5p	OR	6.5	0.6–77.9	19.3:1
hsa-miR-486-5p	OR	3.77	0.79–18.6	19.1:1
hsa-miR-411-3p	OR	3.94	0.81–23.58	17.7:1
hsa-miR-720	FC	0.39	0.12–1.23	17.3:1
hsa-miR-423-5p	FC	2.84	0.8–10.58	17.3:1
hsa-miR-100-5p	FC	0.36	0.1–1.21	17.2:1
hsa-miR-29b-1-5p	FC	2.54	0.73–7.36	16.9:1
hsa-miR-1260a	FC	0.39	0.12–1.27	16.5:1
hsa-miR-1538	FC	3.2	0.78–14.64	16.4:1
hsa-miR-652-3p	OR	0.26	0.04–1.36	15.9:1
hsa-miR-920	OR	0.18	0.02–1.57	15.9:1
hsa-miR-140-5p	OR	3.52	0.7–18.2	15.9:1
hsa-miR-216b	OR	4.17	0.73–33.55	15.3:1
hsa-miR-203	FC	0.37	0.1–1.21	15.1:1
hsa-miR-1182	OR	0.05	0–3.01	14.9:1
hsa-miR-517c-3p	OR	4.2	0.67–32.17	14.9:1
hsa-miR-323a-5p	OR	5.63	0.57–70.71	14.7:1
hsa-miR-125b-5p	FC	0.39	0.11–1.35	14.5:1
hsa-miR-199b-5p	OR	0.04	0–3.45	14.4:1
hsa-miR-630	OR	0.05	0–3.5	14:01
hsa-miR-604	FC	2.48	0.78–8.26	13.7:1
hsa-miR-145-5p	OR	3.31	0.71–18.2	13.6:1
hsa-miR-324-5p	OR	3.23	0.66–14.93	13.6:1
hsa-miR-181b-5p	OR	3.19	0.65–14.98	13.1:1
hsa-miR-146a-3p	OR	3.87	0.61–27.94	13:01
hsa-miR-101-3p	FC	0.31	0.06–1.54	12.7:1
hsa-miR-224-3p	OR	3.35	0.61–18.29	12.4:1
hsa-miR-570-3p	OR	0.2	0.02–2.02	12.3:1
hsa-miR-642a-5p	OR	3.02	0.65–14.76	12.3:1
hsa-miR-19a-3p	FC	0.34	0.08–1.59	12:01
hsa-miR-373-5p	FC	2.42	0.7–7.85	11.8:1
hsa-miR-24-2-5p	OR	3.18	0.64–18.3	11.5:1
hsa-miR-24-3p	FC	0.43	0.13–1.42	11.2:1

CI 95, 95% confidence intervals; POR, posterior odds ratio; FC, fold change; OR, odd ratio.

hsa-miR-489	OR	3.16	0.57–17.38	11:01
hsa-miR-105-3p	FC	0.41	0.12–1.5	10.8:1
hsa-miR-195-3p	OR	2.92	0.59–13	10.7:1
hsa-miR-361-5p	OR	0.06	0–3.61	10.6:1
hsa-miR-892a	OR	0.06	0–3.66	10.5:1
hsa-miR-365a-3p	OR	3.18	0.57–18.26	10.5:1
hsa-miR-138-2-3p	OR	0.06	0–3.21	10.4:1
hsa-miR-429	FC	0.42	0.12–1.44	10.4:1
hsa-miR-20a-3p	OR	0.06	0–4.96	10.4:1
hsa-miR-25-5p	OR	0.06	0–3.33	10.3:1
hsa-miR-873-5p	OR	0.06	0–3.63	10.1:1
hsa-miR-185-3p	FC	0.4	0.1–1.49	10.1:1
hsa-miR-218-5p	OR	3.11	0.59–17.36	10:01
hsa-miR-605	FC	0.46	0.15–1.48	9.6:1
hsa-miR-659-3p	OR	0.06	0–3.05	9.6:1
hsa-miR-330-3p	OR	0.07	0–3.74	9.3:1
hsa-miR-133b	OR	0.07	0–3.43	9.3:1
hsa-miR-23b-3p	FC	0.46	0.14–1.46	9.3:1
hsa-miR-15b-3p	OR	0.07	0–3.7	9.2:1
hsa-miR-758	OR	0.06	0–3.82	9:01
hsa-miR-2113	OR	0.26	0.03–2.23	9:01
hsa-miR-555	FC	0.44	0.12–1.57	8.8:1
hsa-miR-363-3p	OR	0.29	0.04–1.87	8.8:1
hsa-miR-21-5p	FC	0.47	0.13–1.41	8.8:1
hsa-miR-510	OR	2.84	0.56–14.93	8.7:1
hsa-miR-629-5p	OR	2.72	0.54–13.1	8.7:1
hsa-miR-637	OR	0.07	0–3.34	8.6:1
hsa-miR-30a-3p	FC	0.46	0.15–1.55	8.6:1
hsa-miR-593-5p	OR	0.26	0.02–2.14	8.6:1
hsa-miR-1911-3p	OR	0.27	0.03–2.13	8.6:1
hsa-miR-543	FC	2.07	0.67–6.51	8.4:1
hsa-miR-30c-5p	FC	0.45	0.13–1.59	8.4:1
hsa-miR-539-5p	OR	0.27	0.03–2.33	8.4:1
hsa-miR-19b-3p	FC	0.42	0.1–1.7	8:01
hsa-miR-551b-3p	OR	4.32	0.4–64.46	7.8:1
hsa-miR-29a-5p	OR	2.54	0.51–12.07	7.7:1
hsa-miR-511	OR	4.31	0.34–54.36	7.7:1
hsa-miR-519e-5p	OR	4.28	0.33–55.75	7.7:1
hsa-miR-154-5p	OR	4.37	0.36–66.64	7.7:1
hsa-miR-93-5p	FC	0.5	0.17–1.7	7.7:1
hsa-miR-192-5p	FC	2.15	0.63–7.36	7.6:1
hsa-miR-1912	OR	0.34	0.05–2.19	7.6:1
hsa-miR-141-5p	FC	1.99	0.61–5.87	7.6:1
hsa-miR-28-5p	OR	0.37	0.07–1.94	7.5:1
hsa-miR-103b	FC	2.43	0.61–11.35	7.4:1
hsa-miR-1271-5p	OR	4.11	0.32–49.69	7.4:1
hsa-miR-1914-3p	OR	3.34	0.44–29.27	7.4:1
hsa-miR-371a-3p	OR	3.16	0.42–22.57	7.2:1
hsa-miR-548c-5p	OR	2.56	0.5–12.51	7.1:1
hsa-miR-136-5p	OR	3.15	0.47–23.88	7:01

CI 95, 95% confidence intervals; POR, posterior odds ratio; FC, fold change; OR, odd ratio.

hsa-miR-877-3p	FC	1.95	0.64–6.05	7:01
hsa-miR-663b	FC	2.3	0.58–9.91	6.9:1
hsa-miR-26b-3p	OR	4.19	0.36–59.1	6.9:1
hsa-miR-26a-5p	FC	0.51	0.16–1.62	6.9:1
hsa-miR-887	OR	2.47	0.55–12.26	6.9:1
hsa-miR-200a-3p	FC	0.48	0.14–1.72	6.8:1
hsa-miR-154-3p	OR	2.91	0.48–21.82	6.8:1
hsa-miR-639	FC	1.96	0.6–6.08	6.7:1
hsa-miR-183-5p	FC	0.48	0.14–1.82	6.7:1
hsa-miR-18b-5p	FC	0.5	0.15–1.64	6.6:1
hsa-miR-1238	OR	3.07	0.42–22.4	6.6:1
hsa-miR-16-5p	FC	0.48	0.14–1.76	6.6:1
hsa-miR-221-3p	FC	0.45	0.11–1.85	6.6:1
hsa-miR-1183	FC	1.9	0.62–5.81	6.5:1
hsa-miR-654-3p	FC	1.96	0.6–6.39	6.5:1
hsa-miR-10a-5p	FC	0.5	0.14–1.62	6.5:1
hsa-miR-525-3p	OR	3.61	0.34–45.22	6.5:1
hsa-miR-517a-3p	OR	3.64	0.33–43.72	6.4:1
hsa-miR-580	OR	3.68	0.33–40.82	6.4:1
hsa-miR-7-1-3p	OR	2.4	0.53–11.65	6.4:1
hsa-miR-148a-3p	FC	0.42	0.09–2.03	6.4:1
hsa-let-7f-1-3p	OR	3.64	0.28–38.72	6.3:1
hsa-miR-147b	OR	3.58	0.32–41.79	6.3:1
hsa-miR-372	OR	3.7	0.32–52.66	6.3:1
hsa-miR-30e-3p	FC	0.5	0.14–1.63	6.2:1
hsa-miR-144-5p	OR	3.68	0.28–46.17	6.2:1
hsa-miR-298	OR	3.5	0.35–41.5	6.2:1
hsa-miR-512-5p	OR	3.6	0.34–47	6.1:1
hsa-miR-942	OR	0.1	0–5.04	6.1:1
hsa-miR-296-5p	OR	0.09	0–6.16	6.1:1
hsa-miR-196b-3p	OR	0.09	0–4.9	6.1:1
hsa-miR-625-3p	OR	3.65	0.36–49.78	6.1:1
hsa-miR-590-5p	OR	2.57	0.44–15.29	6.1:1
hsa-miR-628-3p	OR	2.57	0.43–15.27	6:01
hsa-let-7g-3p	OR	0.1	0–5.26	6:01
hsa-miR-181a-2-3p	OR	0.39	0.07–2.09	6:01
hsa-miR-662	FC	0.45	0.11–1.93	5.9:1
hsa-miR-638	FC	0.54	0.17–1.65	5.9:1
hsa-miR-141-3p	FC	0.5	0.14–1.75	5.9:1
hsa-miR-487a	OR	3.55	0.31–49.34	5.9:1
hsa-miR-889	OR	0.1	0–5.13	5.8:1
hsa-miR-933	OR	0.09	0–7.67	5.8:1
hsa-miR-374b-3p	OR	0.1	0–5.66	5.8:1
hsa-miR-22-5p	OR	0.1	0–5.25	5.8:1
hsa-miR-27b-3p	FC	0.51	0.14–1.64	5.7:1
hsa-miR-361-3p	FC	0.5	0.13–1.77	5.7:1
hsa-miR-148b-5p	OR	0.1	0–5.72	5.7:1
hsa-miR-888-3p	OR	0.1	0–5.1	5.7:1
hsa-miR-15a-3p	OR	0.1	0–4.81	5.7:1
hsa-miR-1200	OR	0.1	0–5.28	5.6:1

CI 95, 95% confidence intervals; POR, posterior odds ratio; FC, fold change; OR, odd ratio.

hsa-miR-26a-1-3p	OR	0.1	0–5.32	5.6:1
hsa-miR-20b-3p	OR	0.1	0–7.02	5.6:1
hsa-miR-323b-5p	FC	2.06	0.54–8.5	5.6:1
hsa-miR-375	FC	0.53	0.16–1.73	5.6:1
hsa-miR-663a	FC	0.53	0.17–1.79	5.6:1
hsa-miR-592	OR	0.1	0–5.77	5.5:1
hsa-miR-1265	OR	0.1	0–6.4	5.5:1
hsa-let-7a-2-3p	OR	0.1	0–5.39	5.5:1
hsa-miR-296-3p	OR	0.33	0.03–3.09	5.5:1
hsa-miR-556-5p	OR	0.1	0–5.48	5.5:1
hsa-miR-634	OR	0.1	0–6.53	5.5:1
hsa-miR-614	OR	0.1	0–6.48	5.4:1
hsa-miR-135b-3p	OR	0.1	0–6.81	5.4:1
hsa-miR-506-3p	OR	0.1	0–6.6	5.4:1
hsa-miR-520d-3p	FC	0.47	0.11–2.02	5.4:1
hsa-miR-106b-3p	OR	2.41	0.47–14.92	5.3:1
hsa-miR-664-3p	OR	2.16	0.45–9.63	5.2:1
hsa-miR-188-5p	OR	2.36	0.39–13.44	5.2:1
hsa-miR-548i	OR	3.13	0.3–42.22	5:01
hsa-miR-553	OR	0.11	0–6.06	5:01
hsa-miR-636	FC	2.02	0.48–8.03	5:01
hsa-miR-602	FC	0.53	0.16–1.95	5:01
hsa-miR-130b-5p	OR	3	0.29–34.11	5:01
hsa-miR-133a	OR	0.35	0.03–2.92	5:01
hsa-miR-198	OR	2.51	0.36–17.59	5:01
hsa-miR-211-5p	OR	2.54	0.34–17.73	5:01
hsa-miR-616-3p	OR	0.34	0.03–2.75	4.9:1
hsa-miR-370	OR	0.34	0.03–3.07	4.9:1
hsa-miR-134	FC	1.75	0.54–5.55	4.9:1
hsa-miR-615-5p	OR	0.11	0–8.04	4.9:1
hsa-miR-409-5p	OR	0.12	0–7.32	4.9:1
hsa-miR-891b	OR	0.1	0–7.55	4.9:1
hsa-miR-219-1-3p	OR	2.99	0.29–35.96	4.9:1
hsa-miR-1270	OR	3	0.28–31.88	4.8:1
hsa-miR-33b-5p	OR	0.12	0–6.98	4.8:1
hsa-miR-9-3p	OR	2.26	0.38–12.16	4.8:1
hsa-miR-31-5p	FC	0.55	0.16–2.02	4.8:1
hsa-miR-876-5p	OR	0.11	0–6.55	4.8:1
hsa-miR-1256	OR	2.48	0.38–16.79	4.8:1
hsa-miR-34b-3p	OR	0.11	0–7.67	4.8:1
hsa-miR-323a-3p	OR	0.11	0–5.85	4.8:1
hsa-miR-660-5p	FC	0.5	0.13–2.1	4.8:1
hsa-miR-34c-5p	OR	0.12	0–6.45	4.8:1
hsa-miR-455-3p	OR	2.06	0.43–9.58	4.7:1
hsa-miR-601	OR	0.12	0–9.1	4.7:1
hsa-miR-105-5p	OR	0.11	0–7.13	4.7:1
hsa-miR-19b-2-5p	OR	2.1	0.46–10.17	4.7:1
hsa-miR-190b	OR	0.12	0–6	4.7:1
hsa-miR-505-5p	FC	0.51	0.12–2.16	4.7:1
hsa-miR-654-5p	OR	0.12	0–6.89	4.7:1

CI 95, 95% confidence intervals; POR, posterior odds ratio; FC, fold change; OR, odd ratio.

hsa-miR-641	OR	0.12	0–6.42	4.7:1
hsa-miR-513a-5p	OR	0.12	0–6.28	4.6:1
hsa-miR-143-3p	OR	2.12	0.4–10.33	4.6:1
hsa-miR-941	OR	0.12	0–5.21	4.6:1
hsa-miR-501-3p	OR	2.14	0.41–10.71	4.6:1
hsa-miR-151a-5p	FC	0.55	0.16–1.95	4.6:1
hsa-miR-194-3p	OR	2.12	0.37–9.45	4.5:1
hsa-miR-615-3p	FC	0.59	0.19–1.93	4.4:1
hsa-miR-200c-3p	FC	0.59	0.18–1.88	4.3:1
hsa-miR-550a-5p	OR	1.99	0.41–8.98	4.3:1
hsa-miR-223-3p	FC	0.54	0.13–2.14	4.3:1
hsa-miR-1255b-5p	OR	2.17	0.37–11.82	4.3:1
hsa-miR-187-3p	OR	0.48	0.09–2.26	4.2:1
hsa-miR-622	OR	1.99	0.42–9.27	4.2:1
hsa-miR-1972	FC	0.56	0.16–2.14	4.1:1
hsa-miR-545-3p	OR	2.02	0.42–11.12	4.1:1
hsa-miR-532-3p	FC	1.84	0.49–7.44	4.1:1
hsa-miR-99a-5p	FC	0.58	0.18–2.1	4.1:1
hsa-miR-149-5p	FC	0.59	0.16–2.03	4:01
hsa-miR-184	OR	0.39	0.04–3.05	4:01
hsa-miR-491-5p	OR	0.5	0.1–2.63	4:01
hsa-miR-30d-5p	FC	0.59	0.17–1.95	4:01
hsa-let-7f-5p	FC	0.59	0.16–1.98	3.9:1
hsa-miR-299-5p	OR	0.46	0.07–2.87	3.9:1
hsa-miR-182-3p	FC	0.6	0.18–2.04	3.8:1
hsa-miR-1269a	OR	1.91	0.41–8.72	3.8:1
hsa-miR-491-3p	OR	0.42	0.04–3.11	3.8:1
hsa-miR-30c-2-3p	OR	1.87	0.39–8.46	3.8:1
hsa-miR-675-3p	OR	1.92	0.36–8.24	3.8:1
hsa-miR-598	FC	0.6	0.19–2.02	3.7:1
hsa-miR-505-3p	FC	1.86	0.38–7.89	3.7:1
hsa-miR-222-3p	FC	0.6	0.18–2.1	3.7:1
hsa-miR-612	OR	0.47	0.08–3.01	3.7:1
hsa-miR-30c-1-3p	OR	0.41	0.04–3.27	3.6:1
hsa-miR-744-3p	FC	1.56	0.52–4.79	3.6:1
hsa-miR-1914-5p	OR	0.51	0.09–2.83	3.5:1
hsa-miR-376a-3p	OR	2.04	0.33–11.53	3.5:1
hsa-miR-331-5p	FC	0.64	0.22–2.12	3.5:1
hsa-miR-188-3p	FC	0.64	0.21–2.06	3.5:1
hsa-miR-200b-5p	FC	0.59	0.16–2.2	3.5:1
hsa-miR-22-3p	FC	0.6	0.16–2.2	3.5:1
hsa-miR-132-3p	FC	1.57	0.49–5	3.4:1
hsa-miR-10b-5p	FC	0.64	0.2–2.15	3.3:1
hsa-miR-29b-3p	FC	0.61	0.16–2.34	3.3:1
hsa-miR-484	FC	0.64	0.19–2.03	3.3:1
hsa-miR-106b-5p	OR	0.55	0.11–2.69	3.3:1
hsa-miR-558	FC	0.64	0.18–2.18	3.3:1
hsa-miR-152	FC	1.68	0.38–6.53	3.2:1
hsa-miR-106a-5p	FC	0.63	0.17–2.29	3.2:1
hsa-miR-212-3p	FC	1.64	0.38–5.87	3.1:1

CI 95, 95% confidence intervals; POR, posterior odds ratio; FC, fold change; OR, odd ratio.

hsa-miR-1237	OR	0.53	0.08–2.78	3.1:1
hsa-let-7d-3p	FC	0.66	0.2–2.11	3.1:1
hsa-miR-1	OR	1.87	0.33–12.71	3.1:1
hsa-miR-99b-3p	OR	1.68	0.36–7.31	3.1:1
hsa-miR-302d-3p	OR	0.44	0.04–4.12	3.1:1
hsa-miR-1254	OR	1.72	0.36–7.84	3.1:1
hsa-miR-193a-5p	FC	0.64	0.18–2.36	3.1:1
hsa-miR-10b-3p	OR	0.53	0.08–3.27	3.1:1
hsa-miR-425-5p	FC	0.59	0.12–2.57	3.1:1
hsa-miR-425-3p	OR	0.57	0.1–2.97	3.1:1
hsa-miR-339-5p	FC	0.63	0.17–2.58	3.1:1
hsa-miR-455-5p	OR	1.88	0.28–10.94	3:01
hsa-miR-326	OR	0.53	0.08–3.27	3:01
hsa-miR-193b-5p	OR	0.56	0.1–2.86	3:01
hsa-miR-410	OR	2.02	0.23–15.9	3:01
hsa-miR-512-3p	OR	1.86	0.31–12.57	3:01
hsa-miR-376c	OR	1.69	0.37–7.91	3:01
hsa-miR-20b-5p	OR	0.56	0.11–2.99	2.9:1
hsa-miR-382-5p	OR	2.03	0.25–16.69	2.9:1
hsa-miR-10a-3p	OR	1.66	0.37–7.28	2.9:1
hsa-miR-1207-5p	FC	0.68	0.22–2.11	2.9:1
hsa-miR-608	OR	1.69	0.35–8.79	2.9:1
hsa-miR-514a-3p	OR	1.79	0.3–11.68	2.9:1
hsa-miR-27a-3p	FC	0.67	0.19–2.25	2.9:1
hsa-miR-138-5p	OR	0.58	0.1–2.9	2.8:1
hsa-miR-301a-3p	OR	1.79	0.26–11.01	2.8:1
hsa-miR-1468	FC	0.62	0.14–2.77	2.8:1
hsa-miR-192-3p	OR	1.91	0.22–13.89	2.8:1
hsa-miR-562	FC	0.69	0.22–2.16	2.8:1
hsa-miR-205-5p	FC	0.67	0.21–2.53	2.8:1
hsa-miR-518a-3p	OR	1.91	0.25–14.65	2.8:1
hsa-let-7d-5p	FC	0.64	0.15–2.54	2.7:1
hsa-miR-517b-3p	OR	0.21	0–12.09	2.7:1
hsa-miR-550a-3p	OR	1.75	0.29–10.23	2.7:1
hsa-miR-302a-5p	OR	1.69	0.33–9.41	2.7:1
hsa-miR-148a-5p	OR	1.85	0.24–13.99	2.7:1
hsa-miR-155-3p	OR	0.23	0–19.92	2.7:1
hsa-miR-204-5p	FC	0.69	0.21–2.32	2.7:1
hsa-miR-548j	OR	1.85	0.25–15.07	2.7:1
hsa-miR-623	OR	1.72	0.29–10.2	2.7:1
hsa-miR-487b	OR	0.2	0–14.29	2.7:1
hsa-miR-646	OR	1.73	0.3–10.43	2.7:1
hsa-miR-135b-5p	OR	1.58	0.34–7.53	2.7:1
hsa-miR-130b-3p	OR	0.2	0–14.05	2.7:1
hsa-miR-1204	OR	0.21	0–16.26	2.7:1
hsa-miR-600	OR	0.2	0–12.42	2.7:1
hsa-miR-19b-1-5p	OR	0.22	0–15.34	2.7:1
hsa-miR-588	OR	0.2	0–13.65	2.7:1
hsa-miR-450b-5p	OR	0.21	0–16.6	2.6:1
hsa-miR-890	OR	0.2	0–16.9	2.6:1

CI 95, 95% confidence intervals; POR, posterior odds ratio; FC, fold change; OR, odd ratio.

hsa-miR-519a-3p	OR	0.21	0–15.08	2.6:1
hsa-miR-181c-3p	OR	0.21	0–16.47	2.6:1
hsa-miR-494	OR	0.21	0–12.69	2.6:1
hsa-miR-513a-3p	OR	0.21	0–14.63	2.6:1
hsa-miR-93-3p	OR	1.64	0.28–7.72	2.6:1
hsa-miR-1537	OR	0.21	0–15.23	2.6:1
hsa-miR-422a	OR	0.2	0–16.64	2.6:1
hsa-miR-100-3p	OR	0.2	0–15.74	2.6:1
hsa-miR-339-3p	OR	1.66	0.3–8.44	2.6:1
hsa-miR-518c-3p	OR	0.21	0–13.58	2.6:1
hsa-miR-767-5p	OR	0.21	0–13.69	2.6:1
hsa-miR-1236	OR	0.2	0–17.4	2.6:1
hsa-miR-1245a	OR	0.21	0–16.33	2.6:1
hsa-miR-647	OR	0.21	0–15.92	2.6:1
hsa-miR-1185-5p	OR	0.21	0–16.76	2.6:1
hsa-miR-208b	OR	0.2	0–14.1	2.6:1
hsa-miR-520c-3p	OR	0.21	0–14.24	2.6:1
hsa-miR-579	OR	0.22	0–16.35	2.6:1
hsa-miR-202-5p	OR	0.21	0–19.13	2.6:1
hsa-miR-96-3p	OR	0.21	0–12.29	2.6:1
hsa-miR-369-5p	OR	0.22	0–15.64	2.6:1
hsa-miR-518b	OR	0.22	0–16.89	2.6:1
hsa-miR-363-5p	OR	0.21	0–17.57	2.6:1
hsa-miR-516b-5p	OR	0.22	0–13.57	2.6:1
hsa-miR-607	OR	0.21	0–24.15	2.6:1
hsa-miR-921	OR	0.22	0–14.52	2.6:1
hsa-miR-1206	OR	0.2	0–15.71	2.6:1
hsa-miR-302b-3p	OR	0.21	0–16.07	2.6:1
hsa-miR-378a-5p	OR	1.86	0.26–15.12	2.6:1
hsa-miR-377-3p	OR	0.22	0–15.86	2.6:1
hsa-miR-21-3p	OR	0.22	0–14.04	2.6:1
hsa-miR-367-3p	OR	0.21	0–17.62	2.6:1
hsa-miR-302e	OR	0.21	0–15.28	2.6:1
hsa-miR-519e-3p	OR	0.21	0–14.42	2.6:1
hsa-miR-337-3p	OR	0.22	0–15.99	2.6:1
hsa-miR-137	OR	0.21	0–19.02	2.6:1
hsa-miR-521	OR	0.22	0–11.08	2.6:1
hsa-miR-520e	OR	0.21	0–14.05	2.6:1
hsa-miR-520g	OR	0.21	0–16.82	2.6:1
hsa-miR-449a	OR	0.21	0–18	2.6:1
hsa-miR-376a-5p	OR	0.23	0–15.96	2.5:1
hsa-miR-938	OR	0.22	0–10.67	2.5:1
hsa-miR-449b-5p	FC	1.56	0.33–6.55	2.5:1
hsa-miR-1179	OR	0.63	0.12–2.84	2.5:1
hsa-miR-1248	OR	0.21	0–16.18	2.5:1
hsa-miR-145-3p	OR	0.21	0–20.65	2.5:1
hsa-miR-519b-3p	OR	0.23	0–14.25	2.5:1
hsa-miR-183-3p	OR	0.21	0–17.27	2.5:1
hsa-miR-524-3p	OR	0.22	0–17.51	2.5:1
hsa-miR-591	OR	0.22	0–15.76	2.5:1

CI 95, 95% confidence intervals; POR, posterior odds ratio; FC, fold change; OR, odd ratio.

hsa-miR-620	OR	0.21	0–16.13	2.5:1
hsa-miR-675-5p	OR	0.21	0–15.3	2.5:1
hsa-miR-519c-3p	OR	0.21	0–15.98	2.5:1
hsa-miR-578	OR	0.23	0–18.48	2.5:1
hsa-miR-26a-2-3p	OR	0.2	0–15.86	2.5:1
hsa-miR-520d-5p	OR	0.22	0–15.65	2.5:1
hsa-miR-222-5p	OR	0.2	0–16	2.5:1
hsa-miR-135a-5p	OR	0.63	0.13–2.93	2.5:1
hsa-miR-448	OR	0.22	0–13.3	2.5:1
hsa-miR-297	FC	0.73	0.24–2.29	2.5:1
hsa-miR-432-3p	OR	0.22	0–15.95	2.5:1
hsa-miR-191-3p	OR	0.21	0–12.85	2.5:1
hsa-miR-518a-5p	OR	0.21	0–15.23	2.5:1
hsa-miR-302c-3p	OR	0.21	0–17.97	2.5:1
hsa-miR-490-3p	FC	0.72	0.22–2.23	2.5:1
hsa-miR-488-3p	OR	0.22	0–15	2.5:1
hsa-miR-544a	OR	0.22	0–14.7	2.5:1
hsa-miR-586	OR	0.21	0–13.6	2.5:1
hsa-miR-924	OR	0.22	0–16.52	2.5:1
hsa-miR-135a-3p	OR	0.22	0–14.75	2.5:1
hsa-miR-219-5p	OR	0.22	0–20.34	2.5:1
hsa-miR-599	OR	0.21	0–17.42	2.5:1
hsa-miR-450b-3p	FC	1.39	0.4–4.24	2.5:1
hsa-miR-138-1-3p	OR	0.22	0–14.32	2.5:1
hsa-miR-520b	OR	0.21	0–13.93	2.5:1
hsa-miR-610	OR	0.22	0–14.17	2.5:1
hsa-miR-1203	OR	1.64	0.28–9.64	2.5:1
hsa-miR-129-2-3p	OR	1.83	0.22–15.39	2.5:1
hsa-miR-335-3p	OR	0.22	0–14.55	2.5:1
hsa-miR-424-3p	OR	0.22	0–12.43	2.5:1
hsa-miR-362-3p	OR	0.21	0–17.64	2.5:1
hsa-miR-518e-3p	OR	0.22	0–13.27	2.5:1
hsa-miR-299-3p	OR	0.2	0–18.63	2.5:1
hsa-miR-936	OR	0.63	0.13–2.96	2.5:1
hsa-miR-380-3p	OR	0.22	0–17.56	2.5:1
hsa-miR-337-5p	OR	0.21	0–20.94	2.5:1
hsa-miR-130a-5p	OR	0.21	0–19.77	2.5:1
hsa-miR-2053	OR	0.22	0–17.4	2.5:1
hsa-miR-556-3p	OR	0.22	0–14.99	2.5:1
hsa-miR-875-5p	OR	0.22	0–18.16	2.5:1
hsa-miR-1264	OR	0.22	0–12.66	2.5:1
hsa-miR-147a	OR	0.22	0–15.24	2.5:1
hsa-miR-876-3p	OR	0.22	0–15.54	2.5:1
hsa-miR-146b-3p	OR	0.22	0–18.05	2.5:1
hsa-miR-515-3p	OR	0.22	0–17.3	2.5:1
hsa-miR-384	OR	0.21	0–18.87	2.5:1
hsa-miR-1178	OR	0.22	0–15.32	2.4:1
hsa-miR-129-1-3p	OR	0.22	0–15.94	2.4:1
hsa-miR-335-5p	OR	0.63	0.13–3.08	2.4:1
hsa-miR-498	OR	0.23	0–15.62	2.4:1

CI 95, 95% confidence intervals; POR, posterior odds ratio; FC, fold change; OR, odd ratio.

hsa-miR-520a-3p	OR	0.23	0–17.99	2.4:1
hsa-miR-219-2-3p	OR	0.21	0–15.91	2.4:1
hsa-miR-516a-3p	OR	0.22	0–14.61	2.4:1
hsa-miR-541-5p	OR	0.22	0–13.87	2.4:1
hsa-miR-1252	OR	0.24	0–14.39	2.4:1
hsa-miR-340-5p	OR	0.23	0–15.86	2.4:1
hsa-miR-15a-5p	FC	1.52	0.36–6.79	2.4:1
hsa-miR-1263	OR	0.23	0–14.35	2.4:1
hsa-miR-500a-5p	OR	0.64	0.14–3.19	2.4:1
hsa-miR-548h-5p	OR	1.53	0.31–7.16	2.4:1
hsa-miR-29c-5p	OR	1.56	0.33–7.44	2.4:1
hsa-miR-1243	OR	0.23	0–18.58	2.4:1
hsa-miR-200c-5p	OR	0.22	0–18.3	2.4:1
hsa-miR-561-3p	OR	0.22	0–15.34	2.4:1
hsa-miR-626	OR	0.23	0–16.7	2.4:1
hsa-miR-503	OR	1.53	0.29–7.59	2.4:1
hsa-miR-330-5p	OR	1.69	0.19–11.06	2.4:1
hsa-miR-409-3p	OR	1.55	0.29–7.97	2.4:1
hsa-miR-658	OR	1.71	0.2–11.43	2.4:1
hsa-miR-27b-5p	OR	1.91	0.12–25.02	2.4:1
hsa-miR-18b-3p	OR	1.71	0.21–12.37	2.3:1
hsa-miR-300	FC	1.5	0.35–6.84	2.3:1
hsa-miR-542-5p	OR	1.55	0.29–7.72	2.3:1
hsa-miR-765	FC	1.39	0.43–4.58	2.3:1
hsa-let-7a-3p	OR	1.88	0.13–28.02	2.3:1
hsa-miR-23a-5p	OR	1.7	0.22–13.04	2.3:1
hsa-miR-34b-5p	OR	1.54	0.31–8.17	2.3:1
hsa-miR-125b-1-3p	OR	1.73	0.25–14.47	2.3:1
hsa-miR-557	OR	1.67	0.21–12.01	2.3:1
hsa-miR-627	OR	0.54	0.06–5.46	2.3:1
hsa-miR-103a-3p	FC	1.32	0.49–3.54	2.3:1
hsa-miR-541-3p	OR	0.55	0.06–5.7	2.3:1
hsa-miR-769-5p	OR	1.9	0.13–31.68	2.3:1
hsa-miR-769-3p	OR	0.55	0.06–4.77	2.3:1
hsa-miR-515-5p	OR	1.93	0.14–25.5	2.3:1
hsa-miR-582-5p	OR	1.71	0.23–12.46	2.3:1
hsa-miR-1267	OR	1.95	0.15–32.31	2.3:1
hsa-miR-516a-5p	OR	1.91	0.12–28.58	2.3:1
hsa-miR-99a-3p	OR	1.51	0.32–7.44	2.3:1
hsa-let-7g-5p	FC	0.71	0.19–2.6	2.3:1
hsa-miR-127-5p	OR	1.94	0.14–26.95	2.3:1
hsa-miR-492	OR	1.91	0.16–33.61	2.3:1
hsa-miR-1227	OR	0.55	0.06–4.76	2.2:1
hsa-miR-508-3p	OR	1.85	0.13–29.69	2.2:1
hsa-miR-139-5p	FC	0.73	0.19–2.41	2.2:1
hsa-miR-301b	OR	1.89	0.15–31.54	2.2:1
hsa-miR-522-3p	OR	1.88	0.13–25.4	2.2:1
hsa-miR-379-5p	OR	1.87	0.1–23.96	2.2:1
hsa-miR-548d-5p	OR	1.84	0.14–26.84	2.2:1
hsa-miR-574-3p	FC	0.73	0.22–2.33	2.2:1

CI 95, 95% confidence intervals; POR, posterior odds ratio; FC, fold change; OR, odd ratio.

hsa-miR-454-5p	OR	0.68	0.15–3.6	2.2:1
hsa-miR-23b-5p	OR	1.91	0.13–29.12	2.2:1
hsa-miR-583	OR	1.89	0.16–35.26	2.2:1
hsa-miR-1184	OR	0.56	0.05–4.78	2.2:1
hsa-miR-649	OR	1.87	0.13–26.31	2.2:1
hsa-miR-140-3p	FC	1.42	0.38–6.16	2.2:1
hsa-miR-597	OR	1.89	0.12–28.35	2.2:1
hsa-miR-589-3p	OR	1.88	0.15–30.2	2.2:1
hsa-miR-340-3p	OR	0.62	0.09–3.9	2.2:1
hsa-miR-885-5p	OR	1.84	0.15–35.65	2.2:1
hsa-miR-124-5p	OR	1.87	0.15–35.06	2.2:1
hsa-miR-548a-5p	OR	1.87	0.12–26.81	2.2:1
hsa-miR-499a-3p	OR	0.62	0.09–3.76	2.2:1
hsa-miR-185-5p	FC	0.72	0.18–2.79	2.1:1
hsa-miR-7-2-3p	OR	0.61	0.08–3.63	2.1:1
hsa-miR-518d-3p	OR	1.87	0.12–25.28	2.1:1
hsa-miR-532-5p	FC	0.7	0.17–2.87	2.1:1
hsa-miR-518f-5p	FC	0.71	0.16–3.03	2.1:1
hsa-let-7i-5p	FC	0.72	0.18–2.85	2.1:1
hsa-miR-199a-3p	OR	1.87	0.14–31.9	2.1:1
hsa-miR-95	FC	1.43	0.33–6.27	2.1:1
hsa-miR-376b	OR	1.85	0.13–35.36	2.1:1
hsa-miR-20a-5p	FC	0.74	0.19–2.63	2.1:1
hsa-miR-519d	OR	0.6	0.07–5.1	2.1:1
hsa-miR-33b-3p	OR	0.67	0.11–3.76	2.1:1
hsa-miR-518e-5p	OR	0.68	0.11–3.57	2:01
hsa-miR-18a-5p	OR	0.69	0.15–3.73	2:01
hsa-miR-181a-5p	FC	1.36	0.37–5.13	2:01
hsa-miR-564	FC	1.3	0.41–3.99	2:01
hsa-miR-200a-5p	OR	0.68	0.12–3.74	2:01
hsa-miR-103a-2-5p	OR	0.6	0.07–5.47	2:01
hsa-miR-1913	FC	0.78	0.25–2.57	2:01
hsa-miR-9-5p	FC	0.72	0.16–3.12	2:01
hsa-miR-452-3p	OR	1.48	0.26–9.25	2:01
hsa-miR-214-5p	OR	1.7	0.12–24.66	2:01
hsa-miR-650	FC	1.28	0.38–3.93	2:01
hsa-miR-1253	OR	1.69	0.13–24.59	2:01
hsa-miR-526b-3p	OR	1.64	0.11–20.69	1.9:1
hsa-miR-549	OR	1.67	0.13–22.66	1.9:1
hsa-miR-325	OR	1.65	0.13–23.05	1.9:1
hsa-miR-1266	FC	1.27	0.39–3.8	1.9:1
hsa-miR-744-5p	OR	0.72	0.15–3.26	1.9:1
hsa-miR-587	OR	1.67	0.1–21.75	1.9:1
hsa-miR-186-5p	FC	1.31	0.32–4.79	1.9:1
hsa-miR-593-3p	OR	0.72	0.15–3.45	1.9:1
hsa-miR-34a-5p	OR	0.72	0.15–3.42	1.9:1
hsa-miR-380-5p	OR	1.49	0.18–11.07	1.9:1
hsa-miR-1911-5p	OR	1.64	0.12–22.5	1.9:1
hsa-miR-32-5p	OR	1.63	0.12–24.84	1.9:1
hsa-let-7i-3p	OR	1.63	0.12–23.61	1.9:1

CI 95, 95% confidence intervals; POR, posterior odds ratio; FC, fold change; OR, odd ratio.

hsa-miR-223-5p	OR	1.44	0.22–8.67	1.9:1
hsa-miR-331-3p	OR	0.72	0.16–3.63	1.9:1
hsa-miR-191-5p	FC	1.24	0.49–3.19	1.9:1
hsa-miR-502-3p	FC	0.74	0.18–3.26	1.9:1
hsa-miR-802	OR	1.66	0.12–23.47	1.9:1
hsa-miR-524-5p	FC	0.76	0.19–3.04	1.9:1
hsa-miR-421	OR	1.42	0.28–8.07	1.9:1
hsa-miR-518d-5p	OR	1.46	0.18–11.17	1.9:1
hsa-miR-26b-5p	FC	0.76	0.18–3.04	1.9:1
hsa-miR-1296	FC	0.78	0.23–2.9	1.9:1
hsa-miR-423-3p	FC	0.76	0.17–2.81	1.9:1
hsa-miR-28-3p	FC	0.78	0.24–2.59	1.9:1
hsa-miR-125b-2-3p	OR	0.62	0.05–5.33	1.9:1
hsa-miR-208a	OR	1.61	0.1–18.89	1.9:1
hsa-miR-490-5p	OR	1.62	0.11–20.26	1.8:1
hsa-miR-526b-5p	OR	1.59	0.14–25.98	1.8:1
hsa-miR-554	OR	1.6	0.11–22.6	1.8:1
hsa-miR-146b-5p	OR	0.74	0.15–3.73	1.8:1
hsa-miR-631	OR	1.34	0.3–6.12	1.8:1
hsa-miR-943	OR	0.73	0.16–3.6	1.8:1
hsa-miR-449b-3p	OR	1.59	0.11–24.57	1.8:1
hsa-miR-571	FC	0.8	0.27–2.67	1.8:1
hsa-miR-548l	OR	1.62	0.13–25.74	1.8:1
hsa-miR-30b-3p	OR	0.71	0.11–4.66	1.8:1
hsa-miR-217	OR	1.45	0.18–10.24	1.8:1
hsa-miR-509-3-5p	OR	1.56	0.1–22.13	1.8:1
hsa-miR-224-5p	OR	1.45	0.18–11.94	1.8:1
hsa-miR-766-3p	OR	0.75	0.16–3.59	1.8:1
hsa-miR-29b-2-5p	OR	1.33	0.27–6.87	1.8:1
hsa-miR-34c-3p	OR	0.71	0.11–4.23	1.8:1
hsa-miR-342-3p	FC	1.26	0.38–4.74	1.7:1
hsa-miR-501-5p	FC	0.79	0.16–2.99	1.7:1
hsa-miR-25-3p	FC	1.23	0.33–4.27	1.7:1
hsa-miR-937	FC	1.22	0.36–3.94	1.7:1
hsa-miR-362-5p	FC	0.82	0.25–2.83	1.7:1
hsa-miR-216a	OR	0.72	0.11–4.56	1.7:1
hsa-miR-1224-3p	FC	1.27	0.28–5.58	1.7:1
hsa-miR-92b-3p	OR	1.27	0.27–5.95	1.7:1
hsa-miR-551a	OR	0.77	0.16–3.89	1.7:1
hsa-miR-218-1-3p	FC	1.2	0.39–3.91	1.7:1
hsa-miR-142-3p	FC	1.23	0.31–4.93	1.6:1
hsa-miR-1539	FC	1.21	0.34–4.68	1.6:1
hsa-miR-596	OR	0.77	0.12–4.79	1.6:1
hsa-miR-324-3p	FC	0.85	0.28–2.71	1.6:1
hsa-miR-181d	OR	0.76	0.15–3.88	1.6:1
hsa-miR-98	OR	0.76	0.11–5.15	1.5:1
hsa-miR-342-5p	OR	0.78	0.12–3.66	1.5:1
hsa-miR-190a	OR	0.75	0.1–4.88	1.5:1
hsa-miR-643	OR	0.8	0.15–4.43	1.5:1
hsa-miR-151a-3p	FC	0.84	0.26–2.99	1.5:1

CI 95, 95% confidence intervals; POR, posterior odds ratio; FC, fold change; OR, odd ratio.

hsa-miR-589-5p	OR	1.2	0.25–5.94	1.5:1
hsa-miR-485-3p	FC	0.88	0.28–2.65	1.5:1
hsa-miR-29c-3p	FC	1.17	0.3–4.73	1.4:1
hsa-miR-148b-3p	OR	0.82	0.16–4.27	1.4:1
hsa-miR-320b	FC	0.87	0.26–2.7	1.4:1
hsa-miR-888-5p	FC	1.14	0.36–3.86	1.4:1
hsa-miR-496	FC	1.16	0.28–4.72	1.4:1
hsa-miR-196a-5p	FC	0.87	0.23–3.59	1.4:1
hsa-miR-629-3p	FC	0.86	0.22–3.33	1.4:1
hsa-miR-215	FC	1.18	0.3–4.21	1.4:1
hsa-miR-143-5p	OR	1.2	0.22–7.37	1.4:1
hsa-miR-107	FC	0.85	0.2–3.38	1.4:1
hsa-miR-624-5p	OR	0.83	0.15–4.53	1.4:1
hsa-miR-551b-5p	OR	1.18	0.19–8.01	1.4:1
hsa-miR-548k	OR	1.17	0.23–5.35	1.4:1
hsa-miR-584-5p	FC	1.15	0.28–4.73	1.4:1
hsa-miR-517-5p	OR	0.81	0.12–6.49	1.4:1
hsa-miR-576-5p	OR	0.78	0.07–7.58	1.4:1
hsa-miR-30d-3p	FC	0.9	0.25–2.99	1.3:1
hsa-miR-493-3p	OR	0.84	0.15–4.34	1.3:1
hsa-miR-1244	FC	0.88	0.24–3.08	1.3:1
hsa-miR-181c-5p	OR	0.82	0.12–6.54	1.3:1
hsa-miR-182-5p	FC	1.14	0.25–5.01	1.3:1
hsa-let-7f-2-3p	OR	0.79	0.07–6.74	1.3:1
hsa-miR-29a-3p	FC	1.14	0.28–4.72	1.3:1
hsa-miR-374a-5p	OR	1.18	0.18–7.16	1.3:1
hsa-miR-760	FC	0.9	0.26–2.93	1.3:1
hsa-miR-197-3p	FC	0.89	0.25–3.4	1.3:1
hsa-miR-450a-5p	OR	1.14	0.22–5.48	1.3:1
hsa-miR-518c-5p	OR	1.16	0.16–7.85	1.3:1
hsa-miR-31-3p	OR	1.14	0.21–6.6	1.3:1
hsa-miR-99b-5p	FC	1.09	0.3–3.79	1.3:1
hsa-miR-365a-5p	OR	0.8	0.08–8.19	1.3:1
hsa-miR-187-5p	OR	0.8	0.08–8.62	1.3:1
hsa-miR-155-5p	OR	1.14	0.19–5.89	1.3:1
hsa-miR-15b-5p	FC	0.9	0.22–3.82	1.3:1
hsa-let-7e-5p	FC	0.9	0.23–3.4	1.3:1
hsa-miR-628-5p	FC	0.91	0.29–2.95	1.3:1
hsa-miR-412	OR	1.13	0.2–6.4	1.3:1
hsa-miR-17-3p	OR	0.82	0.07–7.68	1.3:1
hsa-miR-486-3p	OR	0.88	0.17–3.85	1.3:1
hsa-miR-611	OR	0.88	0.19–4.23	1.3:1
hsa-miR-653	OR	1.14	0.2–6.16	1.3:1
hsa-miR-33a-3p	OR	1.12	0.2–6.08	1.3:1
hsa-miR-497-5p	OR	1.12	0.24–5.93	1.3:1
hsa-miR-195-5p	OR	1.13	0.2–5.9	1.3:1
hsa-miR-595	FC	0.93	0.29–3.05	1.3:1
hsa-miR-502-5p	FC	0.91	0.3–3.04	1.3:1
hsa-miR-433	FC	1.09	0.31–4.18	1.3:1
hsa-miR-431-5p	OR	1.12	0.2–5.91	1.3:1

CI 95, 95% confidence intervals; POR, posterior odds ratio; FC, fold change; OR, odd ratio.

hsa-miR-1471	FC	1.08	0.35–3.36	1.2:1
hsa-miR-573	OR	0.8	0.07–7.52	1.2:1
hsa-miR-200b-3p	FC	0.93	0.29–3.05	1.2:1
hsa-miR-708-3p	FC	0.9	0.24–3.53	1.2:1
hsa-miR-7-5p	OR	1.11	0.22–5.41	1.2:1
hsa-miR-891a	OR	1.13	0.16–7.4	1.2:1
hsa-miR-346	FC	1.08	0.34–3.47	1.2:1
hsa-miR-1205	OR	1.1	0.2–5.04	1.2:1
hsa-miR-193b-3p	FC	1.11	0.25–4.23	1.2:1
hsa-miR-1208	OR	1.11	0.2–8	1.2:1
hsa-miR-338-3p	OR	1.11	0.16–6.91	1.2:1
hsa-miR-1909-3p	FC	1.07	0.32–3.29	1.2:1
hsa-miR-431-3p	OR	1.08	0.23–5.56	1.2:1
hsa-miR-33a-5p	FC	1.07	0.34–3.51	1.2:1
hsa-miR-214-3p	FC	1.09	0.29–4.57	1.2:1
hsa-miR-877-5p	OR	0.91	0.19–4.46	1.2:1
hsa-miR-194-5p	FC	0.94	0.28–3.33	1.2:1
hsa-miR-371a-5p	OR	1.11	0.16–6.99	1.2:1
hsa-miR-374b-5p	FC	1.08	0.28–4.47	1.2:1
hsa-miR-1249	OR	1.09	0.16–6.57	1.2:1
hsa-miR-552	FC	0.94	0.22–3.94	1.2:1
hsa-miR-566	OR	1.08	0.23–5.53	1.2:1
hsa-miR-632	FC	0.95	0.29–2.78	1.1:1
hsa-miR-196b-5p	OR	1.05	0.22–4.94	1.1:1
hsa-miR-383	OR	0.89	0.08–9.38	1.1:1
hsa-miR-130a-3p	OR	0.92	0.18–4.56	1.1:1
hsa-miR-708-5p	OR	1.06	0.18–5.87	1.1:1
hsa-miR-576-3p	OR	0.9	0.09–9.13	1.1:1
hsa-miR-934	FC	0.96	0.3–3.2	1.1:1
hsa-miR-525-5p	OR	0.9	0.09–9.83	1.1:1
hsa-miR-651	OR	1.02	0.1–11.01	1.1:1
hsa-miR-582-3p	OR	1.04	0.2–5.03	1.1:1
hsa-miR-125a-3p	OR	0.91	0.08–9.14	1.1:1
hsa-miR-146a-5p	FC	1.04	0.22–4.49	1.1:1
hsa-miR-181a-3p	OR	0.92	0.09–9.54	1.1:1
hsa-miR-504	OR	1.01	0.1–9.83	1.1:1
hsa-miR-668	OR	0.9	0.08–8.15	1.1:1
hsa-miR-520f	OR	1.02	0.09–10.17	1.1:1
hsa-miR-922	OR	0.92	0.1–9.49	1.1:1
hsa-miR-338-5p	OR	0.93	0.09–9.42	1.1:1
hsa-miR-128	OR	1.01	0.1–10.51	1.1:1
hsa-miR-92a-1-5p	OR	0.92	0.1–10.17	1.1:1
hsa-miR-106a-3p	OR	1.01	0.09–10.11	1.1:1
hsa-miR-432-5p	OR	0.99	0.1–11.2	1.1:1
hsa-miR-940	FC	0.98	0.3–3.39	1.1:1
hsa-miR-24-1-5p	OR	0.91	0.09–8.99	1.1:1
hsa-miR-493-5p	OR	0.92	0.08–8.4	1.1:1
hsa-miR-92a-2-5p	OR	0.92	0.1–10.17	1.1:1
hsa-miR-488-5p	OR	1.01	0.1–10.12	1.1:1
hsa-miR-499a-5p	OR	0.9	0.09–8.43	1.1:1

CI 95, 95% confidence intervals; POR, posterior odds ratio; FC, fold change; OR, odd ratio.

hsa-miR-206	OR	1.01	0.1–10.59	1.1:1
hsa-miR-454-3p	OR	1.02	0.21–5.2	1.1:1
hsa-miR-661	OR	1.04	0.18–5.94	1.1:1
hsa-miR-451a	OR	0.96	0.19–4.56	1.1:1
hsa-miR-671-3p	OR	0.93	0.08–8.73	1.1:1
hsa-miR-18a-3p	OR	0.92	0.1–9.76	1.1:1
hsa-miR-875-3p	OR	0.94	0.09–8.72	1.1:1
hsa-miR-1258	OR	0.93	0.09–9.19	1:01
hsa-miR-101-5p	OR	0.99	0.1–10.91	1:01
hsa-miR-150-5p	FC	0.97	0.2–4.74	1:01
hsa-miR-619	OR	1.02	0.24–4.83	1:01
hsa-miR-153	OR	1	0.1–11.13	1:01
hsa-miR-378a-3p	FC	0.98	0.29–3.4	1:01
hsa-miR-125a-5p	FC	0.99	0.29–3.6	1:01
hsa-miR-16-2-3p	OR	0.93	0.11–9.81	1:01
hsa-miR-609	OR	0.95	0.09–8.86	1:01
hsa-miR-508-5p	OR	0.93	0.09–9.93	1:01
hsa-miR-671-5p	FC	1	0.3–3.3	1:01
hsa-miR-129-5p	OR	0.95	0.09–8.84	1:01
hsa-miR-509-3p	FC	1.01	0.32–3.41	1:01
hsa-miR-520h	FC	1.01	0.27–3.93	1:01
hsa-miR-621	OR	1	0.15–6.1	1:01
hsa-miR-202-3p	OR	0.98	0.14–6.43	1:01
hsa-miR-513c-5p	OR	0.95	0.08–8.28	1:01
hsa-miR-1272	OR	0.99	0.14–6.29	1:01

CI 95, 95% confidence intervals; POR, posterior odds ratio; FC, fold change; OR, odd ratio.
