# Supplementary material for: Urinary MicroRNA Profiling Predicts the Development of Microalbuminuria in Patients with Type 1 Diabetes
Source: J Clin Med. 2015 Jul 17;4(7):1498–517. doi: 10.3390/jcm4071498 (PMC4519802; doi:10.3390/jcm4071498)
Supplement: Supplementary File 1 [file jcm-04-01498-s002.pdf]

## Supplementary Table S2

| MiRBase_ID | Effect | Size | CI 95 | POR |
|---|---|---|---|---|
| hsa-miR-1247-5p | FC | 12.39 | 4.34–37.14 | 11,999:1 |
| hsa-miR-495 | FC | 56.07 | 17.41–162.69 | 11,999:1 |
| hsa-miR-548o-3p | FC | 11.5 | 3.04–42.64 | 11,999:1 |
| hsa-miR-624-5p | OR | 25.32 | 3.67–223.04 | 11,999:1 |
| hsa-miR-767-3p | OR | 357.33 | 5.39–29,304.54 | 5999:01:00 |
| hsa-miR-373-5p | FC | 0.13 | 0.04–0.41 | 5999:01:00 |
| hsa-miR-223-3p | FC | 8.44 | 2.16–28.04 | 2999:01:00 |
| hsa-miR-920 | OR | 26.12 | 2.24–381.63 | 1499:01:00 |
| hsa-miR-21-5p | FC | 5.66 | 1.95–17.32 | 999:01:00 |
| hsa-miR-203 | FC | 6.55 | 1.97–20.9 | 749:01:00 |
| hsa-miR-577 | FC | 5.58 | 1.87–18.1 | 665.7:1 |
| hsa-miR-378a-3p | FC | 0.19 | 0.05–0.61 | 271.7:1 |
| hsa-miR-19b-3p | FC | 0.1 | 0.02–0.52 | 221.2:1 |
| hsa-miR-23a-3p | FC | 4.45 | 1.38–13.8 | 180.8:1 |
| hsa-miR-154-3p | OR | 0.01 | 0–1.02 | 156.9:1 |
| hsa-miR-186-5p | FC | 0.21 | 0.06–0.75 | 145.3:1 |
| hsa-miR-374a-5p | OR | 0.01 | 0–0.93 | 138.5:1 |
| hsa-miR-551b-5p | OR | 0.01 | 0–1.08 | 135.4:1 |
| hsa-miR-363-3p | OR | 0.08 | 0.01–0.81 | 121.4:1 |
| hsa-miR-517-5p | OR | 13.94 | 1.28–241.78 | 104.3:1 |
| hsa-miR-181c-5p | OR | 13.99 | 1.11–216.03 | 95.8:1 |
| hsa-miR-608 | OR | 6.29 | 1.27–34.74 | 95.8:1 |
| hsa-miR-940 | FC | 0.26 | 0.08–0.83 | 94.2:1 |
| hsa-miR-493-3p | OR | 0.12 | 0.02–0.94 | 91.3:1 |
| hsa-miR-376a-3p | OR | 7.68 | 1.15–61.24 | 77.9:1 |
| hsa-miR-205-5p | FC | 3.87 | 1.23–13.55 | 71.3:1 |
| hsa-miR-1908 | OR | 10.47 | 1.18–152.83 | 64.2:1 |
| hsa-miR-190a | OR | 0.02 | 0–1.49 | 62.2:1 |
| hsa-miR-17-5p | FC | 4.43 | 1.05–17.23 | 58.4:1 |
| hsa-miR-98 | OR | 0.02 | 0–1.61 | 56.1:1 |
| hsa-miR-567 | OR | 15.15 | 1.05–355.07 | 51.2:1 |
| hsa-miR-127-3p | OR | 15.4 | 0.92–329.11 | 49:01:00 |
| hsa-miR-570-3p | OR | 8.18 | 0.93–88.85 | 44.8:1 |
| hsa-miR-424-5p | FC | 0.23 | 0.05–0.89 | 43.1:1 |
| hsa-miR-29b-2-5p | OR | 0.18 | 0.03–1.06 | 42.5:1 |
| hsa-miR-1260a | FC | 0.32 | 0.11–0.99 | 41.9:1 |
| hsa-miR-765 | FC | 3.02 | 1.01–9.74 | 38.5:1 |
| hsa-miR-550a-5p | OR | 4.58 | 0.94–22.19 | 38.2:1 |
| hsa-miR-888-5p | FC | 0.33 | 0.11–0.98 | 36.7:1 |
| hsa-miR-340-3p | OR | 6.43 | 0.94–63.38 | 35.1:1 |
| hsa-miR-1914-3p | OR | 0.03 | 0–1.75 | 34.7:1 |
| hsa-miR-200a-5p | OR | 5.65 | 0.95–41.41 | 34.7:1 |
| hsa-miR-7-2-3p | OR | 6.37 | 0.83–51.57 | 33.9:1 |
| hsa-miR-518e-5p | OR | 5.39 | 0.74–30.93 | 32.1:1 |
| hsa-miR-33b-3p | OR | 5.48 | 0.95–41.99 | 32:01:00 |
| hsa-miR-431-5p | OR | 0.15 | 0.01–1.22 | 31.1:1 |
| hsa-miR-302d-3p | OR | 9.37 | 0.71–144.21 | 30.9:1 |

CI 95, 95% confidence intervals; POR, posterior odds ratio; FC, fold change; OR, odd ratio.

| | | | | |
|---|---|---|---|---|
| hsa-miR-499a-3p | OR | 6.42 | 0.78–51.79 | 30.7:1 |
| hsa-miR-524-5p | FC | 3.35 | 0.94–12.67 | 29.6:1 |
| hsa-miR-452-3p | OR | 5.42 | 0.88–48.27 | 28:01:00 |
| hsa-miR-518d-5p | OR | 7.41 | 0.7–97.39 | 27.2:1 |
| hsa-miR-421 | OR | 4.79 | 0.88–33.22 | 26:01:00 |
| hsa-miR-548c-5p | OR | 4.31 | 0.81–21.81 | 25.7:1 |
| hsa-miR-223-5p | OR | 5.42 | 0.83–47.94 | 24.9:1 |
| hsa-miR-224-5p | OR | 7.05 | 0.63–76.7 | 24.8:1 |
| hsa-miR-217 | OR | 7.37 | 0.7–107.11 | 24.6:1 |
| hsa-miR-380-5p | OR | 7.18 | 0.66–85.54 | 24.3:1 |
| hsa-miR-548c-3p | OR | 6.3 | 0.65–62.6 | 24.3:1 |
| hsa-miR-30e-5p | FC | 3.59 | 0.87–15.65 | 24:01:00 |
| hsa-miR-144-3p | OR | 0.04 | 0–2.37 | 23.6:1 |
| hsa-miR-379-3p | OR | 0.04 | 0–2.24 | 21.3:1 |
| hsa-miR-520h | FC | 0.36 | 0.11–1.23 | 18.7:1 |
| hsa-miR-410 | OR | 0.04 | 0–2.58 | 18.7:1 |
| hsa-miR-450b-3p | FC | 2.59 | 0.87–8.01 | 18.6:1 |
| hsa-miR-545-3p | OR | 0.24 | 0.04–1.51 | 18.5:1 |
| hsa-miR-486-3p | OR | 3.54 | 0.75–18.42 | 18:01 |
| hsa-miR-491-5p | OR | 3.72 | 0.68–19.51 | 17.1:1 |
| hsa-miR-505-5p | FC | 0.33 | 0.09–1.38 | 16.7:1 |
| hsa-miR-505-3p | FC | 0.31 | 0.07–1.33 | 16.6:1 |
| hsa-miR-611 | OR | 3.5 | 0.7–17.28 | 16.4:1 |
| hsa-miR-382-5p | OR | 0.05 | 0–2.7 | 16.3:1 |
| hsa-miR-543 | FC | 0.41 | 0.14–1.35 | 16.2:1 |
| hsa-miR-451a | OR | 3.41 | 0.66–17.81 | 15:01 |
| hsa-miR-485-3p | FC | 0.44 | 0.15–1.24 | 14.8:1 |
| hsa-miR-135b-5p | OR | 3.27 | 0.7–15.58 | 14.5:1 |
| hsa-miR-1914-5p | OR | 3.73 | 0.66–21.85 | 13.6:1 |
| hsa-miR-454-3p | OR | 3.36 | 0.64–17.47 | 13.5:1 |
| hsa-miR-195-3p | OR | 3.12 | 0.7–14.54 | 13.4:1 |
| hsa-miR-320a | FC | 2.27 | 0.77–6.71 | 13.3:1 |
| hsa-miR-125b-2-3p | OR | 0.05 | 0–3.16 | 13.1:1 |
| hsa-miR-616-3p | OR | 4.41 | 0.54–45.14 | 13:01 |
| hsa-miR-130a-3p | OR | 0.3 | 0.05–1.48 | 13:01 |
| hsa-miR-299-5p | OR | 3.94 | 0.53–25.04 | 12.9:1 |
| hsa-miR-612 | OR | 3.93 | 0.58–26.35 | 12.9:1 |
| hsa-miR-370 | OR | 4.45 | 0.62–45.56 | 12.7:1 |
| hsa-miR-582-3p | OR | 3.38 | 0.62–19.12 | 12.7:1 |
| hsa-miR-502-3p | FC | 0.34 | 0.08–1.49 | 12.5:1 |
| hsa-miR-296-3p | OR | 4.45 | 0.61–45.03 | 12.4:1 |
| hsa-miR-1909-3p | FC | 0.46 | 0.16–1.31 | 12.1:1 |
| hsa-miR-661 | OR | 3.47 | 0.61–22.9 | 11.9:1 |
| hsa-miR-199b-5p | OR | 6.06 | 0.43–109.55 | 11.6:1 |
| hsa-miR-302c-5p | OR | 0.23 | 0.02–2.1 | 11.4:1 |
| hsa-miR-133a | OR | 4.31 | 0.6–47.34 | 11.3:1 |
| hsa-miR-141-3p | FC | 0.42 | 0.12–1.47 | 11:01 |
| hsa-let-7e-5p | FC | 0.4 | 0.1–1.41 | 10.8:1 |
| hsa-miR-1468 | FC | 0.36 | 0.08–1.49 | 10.7:1 |
| hsa-miR-630 | OR | 5.8 | 0.44–100.24 | 10.7:1 |

CI 95, 95% confidence intervals; POR, posterior odds ratio; FC, fold change; OR, odd ratio.

| | | | | |
|---|---|---|---|---|
| hsa-miR-708-5p | OR | 3.4 | 0.55–20.93 | 10.7:1 |
| hsa-miR-29c-3p | FC | 0.39 | 0.1–1.46 | 10.6:1 |
| hsa-miR-885-3p | FC | 0.49 | 0.18–1.48 | 10.5:1 |
| hsa-miR-9-3p | OR | 3.14 | 0.54–17.54 | 10.5:1 |
| hsa-miR-197-3p | FC | 0.43 | 0.12–1.39 | 10.5:1 |
| hsa-miR-24-2-5p | OR | 0.32 | 0.05–1.69 | 10.5:1 |
| hsa-miR-551b-3p | OR | 0.06 | 0–3.24 | 10.4:1 |
| hsa-miR-1182 | OR | 5.75 | 0.4–96.24 | 10.1:1 |
| hsa-miR-621 | OR | 3.67 | 0.55–32.17 | 10.1:1 |
| hsa-miR-202-3p | OR | 3.63 | 0.52–26.81 | 10.1:1 |
| hsa-miR-1272 | OR | 3.68 | 0.52–27.06 | 10.1:1 |
| hsa-miR-511 | OR | 0.06 | 0–4.08 | 10:01 |
| hsa-miR-1271-5p | OR | 0.06 | 0–3.67 | 10:01 |
| hsa-miR-409-3p | OR | 0.32 | 0.06–1.83 | 9.9:1 |
| hsa-miR-639 | FC | 0.48 | 0.16–1.38 | 9.9:1 |
| hsa-miR-1972 | FC | 0.41 | 0.1–1.49 | 9.8:1 |
| hsa-miR-1255b-5p | OR | 3.16 | 0.55–18.52 | 9.7:1 |
| hsa-miR-132-5p | OR | 2.92 | 0.63–15.42 | 9.7:1 |
| hsa-miR-34b-5p | OR | 0.31 | 0.05–1.81 | 9.6:1 |
| hsa-miR-154-5p | OR | 0.07 | 0–3.87 | 9.5:1 |
| hsa-miR-128 | OR | 0.07 | 0–3.95 | 9.3:1 |
| hsa-miR-26b-3p | OR | 0.06 | 0–3.96 | 9.3:1 |
| hsa-miR-329 | FC | 2.1 | 0.75–6.89 | 9.2:1 |
| hsa-miR-605 | FC | 0.49 | 0.17–1.44 | 9.2:1 |
| hsa-miR-519e-5p | OR | 0.06 | 0–4.59 | 9.2:1 |
| hsa-miR-432-5p | OR | 0.07 | 0–4.01 | 9.2:1 |
| hsa-miR-488-5p | OR | 0.07 | 0–3.94 | 9.1:1 |
| hsa-miR-15b-5p | FC | 2.44 | 0.62–8.93 | 9:01 |
| hsa-let-7f-2-3p | OR | 4.43 | 0.36–60.09 | 8.9:1 |
| hsa-miR-1256 | OR | 3.3 | 0.52–25.11 | 8.9:1 |
| hsa-miR-504 | OR | 0.07 | 0–4.21 | 8.8:1 |
| hsa-miR-520f | OR | 0.07 | 0–4.26 | 8.8:1 |
| hsa-miR-206 | OR | 0.07 | 0–4.04 | 8.8:1 |
| hsa-miR-483-3p | OR | 0.36 | 0.07–1.78 | 8.8:1 |
| hsa-miR-651 | OR | 0.07 | 0–4.38 | 8.7:1 |
| hsa-miR-106a-3p | OR | 0.07 | 0–5.03 | 8.6:1 |
| hsa-miR-153 | OR | 0.07 | 0–3.62 | 8.6:1 |
| hsa-miR-936 | OR | 2.68 | 0.55–13.11 | 8.4:1 |
| hsa-miR-182-5p | FC | 0.4 | 0.08–1.52 | 8.4:1 |
| hsa-miR-198 | OR | 3.24 | 0.46–22.12 | 8.3:1 |
| hsa-miR-101-5p | OR | 0.07 | 0–4.11 | 8.3:1 |
| hsa-miR-573 | OR | 4.49 | 0.4–70.04 | 8.3:1 |
| hsa-miR-20a-3p | OR | 0.07 | 0–4.05 | 8.2:1 |
| hsa-miR-17-3p | OR | 4.45 | 0.33–56.31 | 8.2:1 |
| hsa-miR-576-5p | OR | 4.52 | 0.38–65.48 | 8.2:1 |
| hsa-miR-616-5p | OR | 2.75 | 0.58–16.21 | 8.1:1 |
| hsa-miR-187-5p | OR | 4.44 | 0.46–69.52 | 8.1:1 |
| hsa-miR-1538 | FC | 0.4 | 0.09–1.79 | 8:01 |
| hsa-miR-211-5p | OR | 3.23 | 0.47–22.48 | 8:01 |
| hsa-miR-330-3p | OR | 0.07 | 0–3.38 | 7.9:1 |

CI 95, 95% confidence intervals; POR, posterior odds ratio; FC, fold change; OR, odd ratio.

| | | | | |
|---|---|---|---|---|
| hsa-miR-365a-5p | OR | 4.56 | 0.37–67.76 | 7.8:1 |
| hsa-miR-758 | OR | 0.08 | 0–4.64 | 7.8:1 |
| hsa-miR-455-3p | OR | 2.47 | 0.53–10.67 | 7.8:1 |
| hsa-miR-15b-3p | OR | 0.08 | 0–3.7 | 7.8:1 |
| hsa-miR-602 | FC | 2.11 | 0.62–7.07 | 7.7:1 |
| hsa-miR-1207-5p | FC | 0.52 | 0.18–1.54 | 7.5:1 |
| hsa-miR-362-5p | FC | 0.5 | 0.16–1.61 | 7.5:1 |
| hsa-miR-133b | OR | 0.08 | 0–4.65 | 7.5:1 |
| hsa-miR-16-5p | FC | 2.03 | 0.65–6.81 | 7.4:1 |
| hsa-miR-770-5p | OR | 0.28 | 0.03–2.3 | 7.4:1 |
| hsa-miR-637 | OR | 0.08 | 0–4.62 | 7.4:1 |
| hsa-miR-19b-2-5p | OR | 2.48 | 0.59–12.22 | 7.4:1 |
| hsa-miR-454-5p | OR | 2.56 | 0.54–13.02 | 7.4:1 |
| hsa-miR-18a-5p | OR | 0.38 | 0.07–2 | 7.3:1 |
| hsa-miR-892a | OR | 0.07 | 0–4.98 | 7.2:1 |
| hsa-miR-92b-3p | OR | 2.47 | 0.54–12.36 | 7.2:1 |
| hsa-miR-1181 | OR | 2.37 | 0.52–10.6 | 6.9:1 |
| hsa-miR-146b-5p | OR | 2.5 | 0.52–13.16 | 6.8:1 |
| hsa-miR-486-5p | OR | 2.36 | 0.56–11.59 | 6.8:1 |
| hsa-miR-300 | FC | 2.18 | 0.53–8.22 | 6.8:1 |
| hsa-miR-219-1-3p | OR | 3.65 | 0.3–42.15 | 6.8:1 |
| hsa-miR-7-1-3p | OR | 2.38 | 0.55–11.52 | 6.7:1 |
| hsa-miR-572 | FC | 1.83 | 0.65–5.69 | 6.6:1 |
| hsa-miR-518f-5p | FC | 0.43 | 0.1–1.93 | 6.6:1 |
| hsa-miR-130b-5p | OR | 3.62 | 0.34–46.48 | 6.6:1 |
| hsa-miR-10b-5p | FC | 1.9 | 0.61–5.85 | 6.6:1 |
| hsa-miR-1912 | OR | 2.69 | 0.46–16.74 | 6.5:1 |
| hsa-miR-943 | OR | 2.51 | 0.5–13.72 | 6.4:1 |
| hsa-miR-665 | FC | 2.08 | 0.56–7.7 | 6.4:1 |
| hsa-miR-548i | OR | 3.61 | 0.31–42.96 | 6.4:1 |
| hsa-miR-302d-5p | OR | 0.42 | 0.08–2.05 | 6.2:1 |
| hsa-miR-1270 | OR | 3.6 | 0.33–43.69 | 6.1:1 |
| hsa-miR-548k | OR | 0.44 | 0.09–2.25 | 5.8:1 |
| hsa-miR-27b-3p | FC | 1.84 | 0.55–5.7 | 5.8:1 |
| hsa-miR-342-5p | OR | 2.44 | 0.45–14.32 | 5.8:1 |
| hsa-miR-181d | OR | 2.46 | 0.46–13.19 | 5.7:1 |
| hsa-miR-571 | FC | 0.57 | 0.2–1.73 | 5.7:1 |
| hsa-miR-589-5p | OR | 0.44 | 0.09–2.11 | 5.7:1 |
| hsa-miR-122-5p | FC | 0.54 | 0.18–1.88 | 5.7:1 |
| hsa-miR-188-3p | FC | 1.75 | 0.61–5 | 5.7:1 |
| hsa-miR-629-5p | OR | 2.26 | 0.46–10.85 | 5.7:1 |
| hsa-miR-139-5p | FC | 1.88 | 0.58–6.03 | 5.6:1 |
| hsa-miR-650 | FC | 0.57 | 0.19–1.67 | 5.6:1 |
| hsa-miR-23b-5p | OR | 0.11 | 0–6.84 | 5.4:1 |
| hsa-miR-593-5p | OR | 2.66 | 0.39–16.98 | 5.4:1 |
| hsa-miR-542-5p | OR | 2.28 | 0.42–10.65 | 5.4:1 |
| hsa-miR-2113 | OR | 2.74 | 0.35–17.78 | 5.4:1 |
| hsa-miR-934 | FC | 1.71 | 0.58–4.92 | 5.4:1 |
| hsa-miR-22-3p | FC | 0.52 | 0.14–1.93 | 5.3:1 |
| hsa-miR-20b-5p | OR | 0.4 | 0.06–2.4 | 5.3:1 |

CI 95, 95% confidence intervals; POR, posterior odds ratio; FC, fold change; OR, odd ratio.

| | | | | |
|---|---|---|---|---|
| hsa-miR-552 | FC | 2.14 | 0.47–8.91 | 5.3:1 |
| hsa-miR-492 | OR | 0.11 | 0–7.58 | 5.2:1 |
| hsa-miR-663a | FC | 0.57 | 0.19–1.72 | 5.2:1 |
| hsa-miR-589-3p | OR | 0.11 | 0–6.46 | 5.2:1 |
| hsa-let-7b-3p | OR | 2.16 | 0.42–9.68 | 5.1:1 |
| hsa-miR-1 | OR | 0.36 | 0.04–2.81 | 5.1:1 |
| hsa-miR-512-3p | OR | 0.35 | 0.04–3.14 | 5.1:1 |
| hsa-miR-1224-3p | FC | 0.43 | 0.07–2.12 | 5.1:1 |
| hsa-miR-425-3p | OR | 0.41 | 0.06–2.4 | 5.1:1 |
| hsa-miR-548a-5p | OR | 0.12 | 0–8.89 | 5.1:1 |
| hsa-miR-376b | OR | 0.11 | 0–4.89 | 5:01 |
| hsa-miR-518d-3p | OR | 0.11 | 0–6.62 | 5:01 |
| hsa-miR-127-5p | OR | 0.11 | 0–7.68 | 5:01 |
| hsa-miR-508-3p | OR | 0.12 | 0–6.23 | 5:01 |
| hsa-miR-515-5p | OR | 0.11 | 0–8.73 | 5:01 |
| hsa-miR-516a-5p | OR | 0.12 | 0–7.7 | 5:01 |
| hsa-miR-597 | OR | 0.12 | 0–7.08 | 5:01 |
| hsa-miR-649 | OR | 0.11 | 0–8.01 | 5:01 |
| hsa-miR-199a-3p | OR | 0.12 | 0–6.51 | 5:01 |
| hsa-miR-769-5p | OR | 0.11 | 0–6.29 | 5:01 |
| hsa-miR-301b | OR | 0.11 | 0–7.65 | 4.9:1 |
| hsa-miR-539-5p | OR | 0.35 | 0.04–3.24 | 4.9:1 |
| hsa-miR-30b-3p | OR | 2.42 | 0.38–14.45 | 4.9:1 |
| hsa-miR-1267 | OR | 0.11 | 0–5.61 | 4.9:1 |
| hsa-miR-583 | OR | 0.11 | 0–6.88 | 4.9:1 |
| hsa-miR-379-5p | OR | 0.12 | 0–5.12 | 4.9:1 |
| hsa-miR-885-5p | OR | 0.12 | 0–5.7 | 4.8:1 |
| hsa-let-7a-3p | OR | 0.11 | 0–8.43 | 4.8:1 |
| hsa-miR-455-5p | OR | 0.37 | 0.05–2.98 | 4.8:1 |
| hsa-miR-105-3p | FC | 0.56 | 0.18–1.87 | 4.8:1 |
| hsa-miR-27b-5p | OR | 0.11 | 0–5.29 | 4.8:1 |
| hsa-miR-192-5p | FC | 0.58 | 0.18–1.82 | 4.7:1 |
| hsa-miR-346 | FC | 1.67 | 0.54–4.74 | 4.7:1 |
| hsa-miR-30e-3p | FC | 1.75 | 0.53–5.77 | 4.7:1 |
| hsa-miR-124-5p | OR | 0.11 | 0–6.05 | 4.7:1 |
| hsa-miR-660-5p | FC | 1.9 | 0.47–7.64 | 4.7:1 |
| hsa-miR-381 | OR | 2.13 | 0.44–10.76 | 4.6:1 |
| hsa-miR-548d-5p | OR | 0.11 | 0–8.38 | 4.6:1 |
| hsa-miR-522-3p | OR | 0.12 | 0–6.58 | 4.6:1 |
| hsa-miR-216a | OR | 2.34 | 0.32–13.18 | 4.5:1 |
| hsa-miR-411-3p | OR | 0.48 | 0.09–2.33 | 4.5:1 |
| hsa-miR-1183 | FC | 1.65 | 0.55–4.62 | 4.5:1 |
| hsa-miR-339-5p | FC | 0.56 | 0.17–1.96 | 4.5:1 |
| hsa-miR-876-5p | OR | 0.12 | 0–6.14 | 4.5:1 |
| hsa-miR-218-5p | OR | 2.12 | 0.43–12.36 | 4.5:1 |
| hsa-miR-34c-3p | OR | 2.34 | 0.35–15 | 4.5:1 |
| hsa-miR-26a-5p | FC | 1.65 | 0.56–5.05 | 4.5:1 |
| hsa-miR-328 | OR | 0.48 | 0.1–2.47 | 4.5:1 |
| hsa-miR-941 | OR | 0.12 | 0–8.37 | 4.5:1 |
| hsa-miR-632 | FC | 0.62 | 0.21–1.77 | 4.5:1 |

CI 95, 95% confidence intervals; POR, posterior odds ratio; FC, fold change; OR, odd ratio.

| | | | | |
|---|---|---|---|---|
| hsa-miR-891b | OR | 0.13 | 0–7.85 | 4.4:1 |
| hsa-miR-96-5p | OR | 2.79 | 0.28–26.72 | 4.4:1 |
| hsa-miR-34c-5p | OR | 0.13 | 0–6.25 | 4.4:1 |
| hsa-miR-489 | OR | 2.08 | 0.36–9.88 | 4.4:1 |
| hsa-miR-409-5p | OR | 0.12 | 0–8.56 | 4.3:1 |
| hsa-miR-190b | OR | 0.12 | 0–9.11 | 4.3:1 |
| hsa-miR-513a-5p | OR | 0.12 | 0–6.91 | 4.3:1 |
| hsa-miR-1296 | FC | 1.73 | 0.51–5.74 | 4.3:1 |
| hsa-miR-654-5p | OR | 0.12 | 0–10.02 | 4.3:1 |
| hsa-miR-641 | OR | 0.12 | 0–10.49 | 4.3:1 |
| hsa-miR-615-5p | OR | 0.13 | 0–7.27 | 4.3:1 |
| hsa-miR-33b-5p | OR | 0.13 | 0–7.71 | 4.3:1 |
| hsa-miR-34a-3p | OR | 2.75 | 0.28–27.17 | 4.3:1 |
| hsa-miR-365a-3p | OR | 2.11 | 0.41–10.88 | 4.3:1 |
| hsa-miR-601 | OR | 0.13 | 0–9.58 | 4.2:1 |
| hsa-miR-323a-3p | OR | 0.13 | 0–11.66 | 4.2:1 |
| hsa-miR-662 | FC | 1.89 | 0.45–7.47 | 4.2:1 |
| hsa-miR-553 | OR | 0.12 | 0–9.87 | 4.2:1 |
| hsa-miR-200b-3p | FC | 0.61 | 0.2–1.93 | 4.2:1 |
| hsa-miR-187-3p | OR | 1.98 | 0.42–9.42 | 4.2:1 |
| hsa-miR-1913 | FC | 0.61 | 0.21–1.88 | 4.2:1 |
| hsa-miR-34b-3p | OR | 0.13 | 0–8.49 | 4.1:1 |
| hsa-miR-496 | FC | 0.52 | 0.13–2.32 | 4.1:1 |
| hsa-miR-1203 | OR | 2.12 | 0.36–12.16 | 4.1:1 |
| hsa-miR-199a-5p | OR | 1.96 | 0.41–9.46 | 4.1:1 |
| hsa-miR-215 | FC | 0.59 | 0.17–1.96 | 4:01 |
| hsa-miR-1539 | FC | 1.75 | 0.5–6.95 | 4:01 |
| hsa-miR-324-3p | FC | 1.59 | 0.53–4.61 | 3.9:1 |
| hsa-miR-105-5p | OR | 0.13 | 0–7.97 | 3.9:1 |
| hsa-miR-622 | OR | 0.51 | 0.1–2.43 | 3.9:1 |
| hsa-let-7a-5p | FC | 1.65 | 0.55–5.76 | 3.9:1 |
| hsa-miR-1184 | OR | 2.3 | 0.29–20.49 | 3.9:1 |
| hsa-miR-125a-5p | FC | 0.61 | 0.19–1.95 | 3.9:1 |
| hsa-miR-375 | FC | 0.63 | 0.2–1.86 | 3.9:1 |
| hsa-miR-617 | OR | 0.48 | 0.09–2.98 | 3.9:1 |
| hsa-miR-877-5p | OR | 1.88 | 0.41–8.66 | 3.8:1 |
| hsa-miR-106b-5p | OR | 0.5 | 0.09–2.65 | 3.8:1 |
| hsa-miR-29b-3p | FC | 0.58 | 0.17–2.01 | 3.8:1 |
| hsa-miR-627 | OR | 2.37 | 0.28–22.86 | 3.8:1 |
| hsa-miR-1227 | OR | 2.35 | 0.29–21.08 | 3.8:1 |
| hsa-miR-99b-5p | FC | 0.63 | 0.2–1.95 | 3.8:1 |
| hsa-miR-636 | FC | 1.78 | 0.43–7.16 | 3.7:1 |
| hsa-miR-376c | OR | 1.82 | 0.41–8.19 | 3.7:1 |
| hsa-miR-99b-3p | OR | 1.83 | 0.4–8.62 | 3.7:1 |
| hsa-miR-142-3p | FC | 1.72 | 0.44–6.97 | 3.7:1 |
| hsa-miR-324-5p | OR | 0.55 | 0.12–2.52 | 3.6:1 |
| hsa-miR-10a-3p | OR | 1.83 | 0.43–8.95 | 3.6:1 |
| hsa-miR-541-3p | OR | 2.29 | 0.29–18.55 | 3.5:1 |
| hsa-miR-182-3p | FC | 1.6 | 0.5–5.2 | 3.5:1 |
| hsa-miR-769-3p | OR | 2.23 | 0.26–18.51 | 3.5:1 |

CI 95, 95% confidence intervals; POR, posterior odds ratio; FC, fold change; OR, odd ratio.

| | | | | |
|---|---|---|---|---|
| hsa-miR-1254 | OR | 1.81 | 0.41–8.42 | 3.5:1 |
| hsa-miR-604 | FC | 0.66 | 0.22–1.89 | 3.5:1 |
| hsa-miR-145-5p | OR | 0.56 | 0.12–2.61 | 3.5:1 |
| hsa-miR-10a-5p | FC | 1.55 | 0.49–4.72 | 3.4:1 |
| hsa-miR-146a-3p | OR | 1.98 | 0.35–12.19 | 3.4:1 |
| hsa-miR-26b-5p | FC | 1.62 | 0.45–5.56 | 3.4:1 |
| hsa-miR-642a-5p | OR | 1.77 | 0.38–7.76 | 3.4:1 |
| hsa-miR-425-5p | FC | 1.75 | 0.43–7.72 | 3.3:1 |
| hsa-miR-218-1-3p | FC | 0.65 | 0.22–2.11 | 3.3:1 |
| hsa-let-7d-5p | FC | 1.67 | 0.44–7.29 | 3.3:1 |
| hsa-miR-140-3p | FC | 0.59 | 0.15–2.45 | 3.3:1 |
| hsa-miR-200a-3p | FC | 1.52 | 0.46–4.67 | 3.3:1 |
| hsa-miR-150-5p | FC | 0.57 | 0.13–2.57 | 3.3:1 |
| hsa-miR-136-5p | OR | 0.45 | 0.05–3.95 | 3.3:1 |
| hsa-miR-371a-3p | OR | 0.46 | 0.05–3.63 | 3.3:1 |
| hsa-miR-1238 | OR | 0.46 | 0.05–3.53 | 3.3:1 |
| hsa-miR-619 | OR | 1.79 | 0.37–8.31 | 3.3:1 |
| hsa-miR-342-3p | FC | 0.64 | 0.2–2.18 | 3.3:1 |
| hsa-miR-93-5p | FC | 0.67 | 0.23–2.04 | 3.2:1 |
| hsa-miR-411-5p | OR | 1.72 | 0.39–7.87 | 3.2:1 |
| hsa-miR-658 | OR | 2.05 | 0.3–18.48 | 3.2:1 |
| hsa-miR-558 | FC | 1.54 | 0.46–4.92 | 3.2:1 |
| hsa-miR-193b-5p | OR | 1.82 | 0.36–9.6 | 3.2:1 |
| hsa-miR-30c-2-3p | OR | 1.71 | 0.37–8.14 | 3.2:1 |
| hsa-miR-126-5p | OR | 1.73 | 0.36–7.5 | 3.2:1 |
| hsa-miR-107 | FC | 0.6 | 0.15–2.49 | 3.1:1 |
| hsa-miR-125b-1-3p | OR | 2.08 | 0.24–17.66 | 3.1:1 |
| hsa-miR-548b-3p | OR | 0.5 | 0.07–3.45 | 3.1:1 |
| hsa-miR-23a-5p | OR | 2.06 | 0.28–17.18 | 3.1:1 |
| hsa-miR-582-5p | OR | 2.11 | 0.27–16.82 | 3.1:1 |
| hsa-miR-18b-3p | OR | 2.05 | 0.25–16.82 | 3.1:1 |
| hsa-miR-490-3p | FC | 1.47 | 0.49–4.4 | 3.1:1 |
| hsa-miR-557 | OR | 2.06 | 0.25–16.71 | 3.1:1 |
| hsa-miR-675-3p | OR | 1.71 | 0.35–7.28 | 3.1:1 |
| hsa-miR-302a-5p | OR | 0.53 | 0.08–2.93 | 3.1:1 |
| hsa-miR-30d-3p | FC | 1.5 | 0.45–4.63 | 3:01 |
| hsa-miR-23b-3p | FC | 1.48 | 0.48–4.27 | 3:01 |
| hsa-miR-339-3p | OR | 0.54 | 0.09–3.02 | 3:01 |
| hsa-miR-1269a | OR | 1.69 | 0.39–7.86 | 3:01 |
| hsa-miR-330-5p | OR | 2.01 | 0.24–15.88 | 3:01 |
| hsa-miR-1205 | OR | 1.73 | 0.37–9.34 | 3:01 |
| hsa-miR-18b-5p | FC | 1.47 | 0.46–4.48 | 3:01 |
| hsa-miR-92b-5p | OR | 1.68 | 0.36–7.14 | 3:01 |
| hsa-miR-638 | FC | 0.7 | 0.25–1.98 | 2.9:1 |
| hsa-let-7d-3p | FC | 0.69 | 0.24–2.06 | 2.9:1 |
| hsa-miR-140-5p | OR | 1.66 | 0.37–7.43 | 2.9:1 |
| hsa-miR-518c-5p | OR | 0.49 | 0.05–4.08 | 2.9:1 |
| hsa-miR-431-3p | OR | 1.69 | 0.35–9.04 | 2.8:1 |
| hsa-miR-891a | OR | 0.5 | 0.06–3.85 | 2.8:1 |
| hsa-miR-566 | OR | 1.7 | 0.31–8.08 | 2.8:1 |

CI 95, 95% confidence intervals; POR, posterior odds ratio; FC, fold change; OR, odd ratio.

| | | | | |
|---|---|---|---|---|
| hsa-miR-143-3p | OR | 1.61 | 0.32–8.11 | 2.8:1 |
| hsa-miR-645 | OR | 0.55 | 0.09–3.3 | 2.8:1 |
| hsa-miR-142-5p | OR | 0.6 | 0.13–3.35 | 2.7:1 |
| hsa-miR-584-5p | FC | 0.66 | 0.17–2.39 | 2.7:1 |
| hsa-miR-7-5p | OR | 1.67 | 0.34–8.69 | 2.7:1 |
| hsa-miR-30c-1-3p | OR | 0.51 | 0.05–4.23 | 2.7:1 |
| hsa-miR-491-3p | OR | 0.49 | 0.05–4.78 | 2.7:1 |
| hsa-miR-615-3p | FC | 0.71 | 0.23–1.99 | 2.7:1 |
| hsa-miR-423-5p | FC | 0.69 | 0.19–2.31 | 2.7:1 |
| hsa-miR-592 | OR | 2.2 | 0.14–37.27 | 2.7:1 |
| hsa-miR-614 | OR | 2.19 | 0.14–32.42 | 2.7:1 |
| hsa-miR-374b-3p | OR | 2.18 | 0.14–35.91 | 2.6:1 |
| hsa-miR-942 | OR | 2.2 | 0.13–34.48 | 2.6:1 |
| hsa-miR-148b-5p | OR | 2.16 | 0.13–34.38 | 2.6:1 |
| hsa-miR-888-3p | OR | 2.21 | 0.15–36.71 | 2.6:1 |
| hsa-miR-26a-1-3p | OR | 2.21 | 0.16–36.86 | 2.6:1 |
| hsa-miR-194-3p | OR | 1.62 | 0.32–7.87 | 2.6:1 |
| hsa-miR-501-3p | OR | 1.59 | 0.32–7.53 | 2.6:1 |
| hsa-miR-634 | OR | 2.2 | 0.14–33.43 | 2.6:1 |
| hsa-miR-603 | FC | 1.39 | 0.49–4.2 | 2.6:1 |
| hsa-miR-889 | OR | 2.15 | 0.13–34.58 | 2.6:1 |
| hsa-miR-331-5p | FC | 0.73 | 0.24–2.08 | 2.6:1 |
| hsa-miR-21-3p | OR | 0.22 | 0–22.41 | 2.6:1 |
| hsa-miR-556-5p | OR | 2.1 | 0.15–33.71 | 2.5:1 |
| hsa-let-7a-2-3p | OR | 2.17 | 0.15–38.44 | 2.5:1 |
| hsa-miR-675-5p | OR | 0.21 | 0–12.3 | 2.5:1 |
| hsa-miR-22-5p | OR | 2.15 | 0.14–34.68 | 2.5:1 |
| hsa-miR-323a-5p | OR | 1.8 | 0.23–13.42 | 2.5:1 |
| hsa-miR-518a-5p | OR | 0.24 | 0–15.36 | 2.5:1 |
| hsa-miR-578 | OR | 0.22 | 0–25.37 | 2.5:1 |
| hsa-miR-20b-3p | OR | 2.12 | 0.13–31.89 | 2.5:1 |
| hsa-miR-96-3p | OR | 0.23 | 0–16.86 | 2.5:1 |
| hsa-miR-129-1-3p | OR | 0.23 | 0–15.23 | 2.5:1 |
| hsa-miR-363-5p | OR | 0.22 | 0–16.13 | 2.5:1 |
| hsa-miR-196b-3p | OR | 2.09 | 0.12–33.63 | 2.5:1 |
| hsa-miR-1178 | OR | 0.21 | 0–13.34 | 2.5:1 |
| hsa-miR-449a | OR | 0.23 | 0–14.06 | 2.5:1 |
| hsa-miR-521 | OR | 0.23 | 0–18.54 | 2.5:1 |
| hsa-miR-541-5p | OR | 0.22 | 0–18.93 | 2.5:1 |
| hsa-miR-1265 | OR | 2.15 | 0.14–35.48 | 2.5:1 |
| hsa-miR-362-3p | OR | 0.22 | 0–16.87 | 2.5:1 |
| hsa-miR-607 | OR | 0.23 | 0–22.61 | 2.5:1 |
| hsa-miR-130a-5p | OR | 0.23 | 0–17.53 | 2.5:1 |
| hsa-miR-15a-3p | OR | 2.17 | 0.14–33.31 | 2.5:1 |
| hsa-miR-1200 | OR | 2.14 | 0.13–34.11 | 2.5:1 |
| hsa-miR-181c-3p | OR | 0.23 | 0–17.18 | 2.5:1 |
| hsa-miR-296-5p | OR | 2.17 | 0.14–37.73 | 2.5:1 |
| hsa-miR-191-3p | OR | 0.23 | 0–14.81 | 2.5:1 |
| hsa-miR-1204 | OR | 0.22 | 0–14.63 | 2.5:1 |
| hsa-miR-654-3p | FC | 1.39 | 0.47–4.37 | 2.5:1 |

CI 95, 95% confidence intervals; POR, posterior odds ratio; FC, fold change; OR, odd ratio.

| | | | | |
|---|---|---|---|---|
| hsa-miR-145-3p | OR | 0.23 | 0–18.11 | 2.5:1 |
| hsa-miR-620 | OR | 0.22 | 0–19.59 | 2.5:1 |
| hsa-miR-506-3p | OR | 2.13 | 0.14–35.74 | 2.5:1 |
| hsa-miR-424-3p | OR | 0.22 | 0–17.57 | 2.5:1 |
| hsa-miR-519b-3p | OR | 0.22 | 0–13.66 | 2.5:1 |
| hsa-miR-337-5p | OR | 0.22 | 0–16.44 | 2.5:1 |
| hsa-let-7c | FC | 1.38 | 0.43–4.29 | 2.5:1 |
| hsa-let-7g-3p | OR | 2.1 | 0.15–37.94 | 2.5:1 |
| hsa-miR-1236 | OR | 0.22 | 0–16.1 | 2.5:1 |
| hsa-miR-135a-3p | OR | 0.22 | 0–19.45 | 2.5:1 |
| hsa-miR-450b-5p | OR | 0.22 | 0–17.6 | 2.5:1 |
| hsa-miR-933 | OR | 2.09 | 0.14–32.4 | 2.5:1 |
| hsa-miR-147a | OR | 0.23 | 0–17.31 | 2.5:1 |
| hsa-miR-519e-3p | OR | 0.23 | 0–20.85 | 2.5:1 |
| hsa-miR-502-5p | FC | 0.73 | 0.25–2.06 | 2.5:1 |
| hsa-miR-219-5p | OR | 0.23 | 0–15.02 | 2.5:1 |
| hsa-miR-579 | OR | 0.22 | 0–21.35 | 2.5:1 |
| hsa-miR-135b-3p | OR | 2.14 | 0.16–37.75 | 2.5:1 |
| hsa-miR-151a-3p | FC | 1.4 | 0.45–4.89 | 2.4:1 |
| hsa-miR-1185-5p | OR | 0.23 | 0–15.19 | 2.4:1 |
| hsa-miR-1248 | OR | 0.22 | 0–14.49 | 2.4:1 |
| hsa-miR-412 | OR | 1.59 | 0.29–9.49 | 2.4:1 |
| hsa-miR-448 | OR | 0.23 | 0–14.5 | 2.4:1 |
| hsa-miR-137 | OR | 0.23 | 0–17.01 | 2.4:1 |
| hsa-miR-19b-1-5p | OR | 0.22 | 0–17.58 | 2.4:1 |
| hsa-miR-513a-3p | OR | 0.22 | 0–13.76 | 2.4:1 |
| hsa-miR-520g | OR | 0.22 | 0–22.78 | 2.4:1 |
| hsa-miR-146b-3p | OR | 0.22 | 0–15.75 | 2.4:1 |
| hsa-miR-515-3p | OR | 0.23 | 0–15.16 | 2.4:1 |
| hsa-miR-337-3p | OR | 0.22 | 0–17.18 | 2.4:1 |
| hsa-miR-488-3p | OR | 0.23 | 0–15.1 | 2.4:1 |
| hsa-miR-518e-3p | OR | 0.23 | 0–12.65 | 2.4:1 |
| hsa-miR-520a-3p | OR | 0.24 | 0–19.88 | 2.4:1 |
| hsa-miR-876-3p | OR | 0.24 | 0–14.76 | 2.4:1 |
| hsa-miR-302b-3p | OR | 0.22 | 0–26 | 2.4:1 |
| hsa-miR-516a-3p | OR | 0.22 | 0–17.57 | 2.4:1 |
| hsa-miR-890 | OR | 0.24 | 0–19.09 | 2.4:1 |
| hsa-miR-369-5p | OR | 0.24 | 0–17.3 | 2.4:1 |
| hsa-miR-610 | OR | 0.23 | 0–15.82 | 2.4:1 |
| hsa-miR-1245a | OR | 0.23 | 0–16.33 | 2.4:1 |
| hsa-miR-335-3p | OR | 0.23 | 0–17.27 | 2.4:1 |
| hsa-miR-1252 | OR | 0.22 | 0–14.01 | 2.4:1 |
| hsa-miR-432-3p | OR | 0.23 | 0–18.02 | 2.4:1 |
| hsa-miR-586 | OR | 0.22 | 0–16.87 | 2.4:1 |
| hsa-miR-26a-2-3p | OR | 0.22 | 0–20.28 | 2.4:1 |
| hsa-miR-299-3p | OR | 0.22 | 0–15.64 | 2.4:1 |
| hsa-miR-524-3p | OR | 0.22 | 0–19.81 | 2.4:1 |
| hsa-miR-556-3p | OR | 0.23 | 0–24.84 | 2.4:1 |
| hsa-miR-600 | OR | 0.22 | 0–15.44 | 2.4:1 |
| hsa-miR-767-5p | OR | 0.23 | 0–11.95 | 2.4:1 |

CI 95, 95% confidence intervals; POR, posterior odds ratio; FC, fold change; OR, odd ratio.

| | | | | |
|---|---|---|---|---|
| hsa-miR-340-5p | OR | 0.23 | 0–15.7 | 2.4:1 |
| hsa-miR-422a | OR | 0.23 | 0–18.14 | 2.4:1 |
| hsa-miR-520e | OR | 0.22 | 0–20.21 | 2.4:1 |
| hsa-miR-33a-3p | OR | 1.6 | 0.29–9.44 | 2.4:1 |
| hsa-miR-1264 | OR | 0.23 | 0–21.71 | 2.4:1 |
| hsa-miR-302e | OR | 0.23 | 0–15.03 | 2.4:1 |
| hsa-miR-626 | OR | 0.23 | 0–13.75 | 2.4:1 |
| hsa-miR-938 | OR | 0.22 | 0–13.61 | 2.4:1 |
| hsa-miR-202-5p | OR | 0.22 | 0–18.59 | 2.4:1 |
| hsa-miR-544a | OR | 0.23 | 0–13.37 | 2.4:1 |
| hsa-miR-516b-5p | OR | 0.23 | 0–20.37 | 2.4:1 |
| hsa-miR-219-2-3p | OR | 0.23 | 0–14.23 | 2.4:1 |
| hsa-miR-1537 | OR | 0.22 | 0–16.13 | 2.4:1 |
| hsa-miR-384 | OR | 0.23 | 0–14.45 | 2.4:1 |
| hsa-miR-520c-3p | OR | 0.25 | 0–13.31 | 2.4:1 |
| hsa-miR-875-5p | OR | 0.23 | 0–13.76 | 2.4:1 |
| hsa-miR-380-3p | OR | 0.23 | 0–20.56 | 2.4:1 |
| hsa-miR-591 | OR | 0.23 | 0–15.56 | 2.4:1 |
| hsa-miR-647 | OR | 0.23 | 0–23.36 | 2.4:1 |
| hsa-miR-214-3p | FC | 0.7 | 0.19–2.44 | 2.4:1 |
| hsa-miR-599 | OR | 0.23 | 0–13.26 | 2.4:1 |
| hsa-miR-377-3p | OR | 0.23 | 0–16.22 | 2.4:1 |
| hsa-miR-518b | OR | 0.23 | 0–19.45 | 2.4:1 |
| hsa-miR-520b | OR | 0.24 | 0–19.98 | 2.4:1 |
| hsa-miR-194-5p | FC | 1.38 | 0.41–4.41 | 2.4:1 |
| hsa-miR-1243 | OR | 0.23 | 0–16.78 | 2.4:1 |
| hsa-miR-487b | OR | 0.23 | 0–17.92 | 2.4:1 |
| hsa-miR-588 | OR | 0.23 | 0–16.66 | 2.4:1 |
| hsa-miR-518c-3p | OR | 0.22 | 0–14.31 | 2.4:1 |
| hsa-miR-523-3p | OR | 1.52 | 0.34–7.37 | 2.4:1 |
| hsa-miR-561-3p | OR | 0.23 | 0–16.28 | 2.3:1 |
| hsa-miR-498 | OR | 0.24 | 0–11.82 | 2.3:1 |
| hsa-miR-574-3p | FC | 0.75 | 0.25–2.22 | 2.3:1 |
| hsa-miR-924 | OR | 0.25 | 0–16.18 | 2.3:1 |
| hsa-miR-183-3p | OR | 0.23 | 0–18.62 | 2.3:1 |
| hsa-miR-222-5p | OR | 0.23 | 0–14.35 | 2.3:1 |
| hsa-miR-517b-3p | OR | 0.23 | 0–17.81 | 2.3:1 |
| hsa-miR-921 | OR | 0.22 | 0–17.42 | 2.3:1 |
| hsa-miR-1263 | OR | 0.22 | 0–18.32 | 2.3:1 |
| hsa-miR-302c-3p | OR | 0.23 | 0–12.34 | 2.3:1 |
| hsa-miR-376a-5p | OR | 0.23 | 0–13.89 | 2.3:1 |
| hsa-miR-494 | OR | 0.22 | 0–18.16 | 2.3:1 |
| hsa-miR-2053 | OR | 0.23 | 0–16.44 | 2.3:1 |
| hsa-miR-155-5p | OR | 1.57 | 0.26–8.23 | 2.3:1 |
| hsa-miR-155-3p | OR | 0.23 | 0–18.6 | 2.3:1 |
| hsa-miR-138-1-3p | OR | 0.22 | 0–20.72 | 2.3:1 |
| hsa-miR-367-3p | OR | 0.23 | 0–17.44 | 2.3:1 |
| hsa-miR-130b-3p | OR | 0.24 | 0–14.05 | 2.3:1 |
| hsa-miR-653 | OR | 1.58 | 0.3–9 | 2.3:1 |
| hsa-miR-210 | FC | 0.69 | 0.18–2.86 | 2.3:1 |

CI 95, 95% confidence intervals; POR, posterior odds ratio; FC, fold change; OR, odd ratio.

| | | | | |
|---|---|---|---|---|
| hsa-miR-100-3p | OR | 0.24 | 0–17.97 | 2.3:1 |
| hsa-miR-519a-3p | OR | 0.25 | 0–17.43 | 2.3:1 |
| hsa-miR-519c-3p | OR | 0.23 | 0–18.15 | 2.3:1 |
| hsa-miR-31-3p | OR | 1.59 | 0.29–8.98 | 2.3:1 |
| hsa-miR-374b-5p | FC | 1.44 | 0.4–5.82 | 2.3:1 |
| hsa-miR-520d-5p | OR | 0.24 | 0–10.84 | 2.3:1 |
| hsa-miR-503 | OR | 0.66 | 0.12–3.09 | 2.3:1 |
| hsa-miR-555 | FC | 1.36 | 0.38–4.54 | 2.3:1 |
| hsa-miR-208b | OR | 0.24 | 0–18.22 | 2.3:1 |
| hsa-miR-452-5p | FC | 1.41 | 0.37–5.41 | 2.3:1 |
| hsa-miR-1206 | OR | 0.25 | 0–15.91 | 2.3:1 |
| hsa-miR-195-5p | OR | 1.57 | 0.27–8.57 | 2.3:1 |
| hsa-miR-99a-3p | OR | 0.65 | 0.14–3.37 | 2.3:1 |
| hsa-miR-200c-5p | OR | 0.24 | 0–14.91 | 2.3:1 |
| hsa-miR-433 | FC | 0.73 | 0.21–2.69 | 2.3:1 |
| hsa-miR-193a-5p | FC | 1.36 | 0.4–4.49 | 2.2:1 |
| hsa-miR-29c-5p | OR | 0.66 | 0.13–3.2 | 2.2:1 |
| hsa-miR-628-5p | FC | 1.31 | 0.43–4.03 | 2.2:1 |
| hsa-miR-1179 | OR | 1.46 | 0.32–7.07 | 2.2:1 |
| hsa-miR-92a-3p | FC | 0.76 | 0.24–2.27 | 2.2:1 |
| hsa-miR-548h-5p | OR | 0.66 | 0.13–3.22 | 2.2:1 |
| hsa-miR-500a-5p | OR | 1.46 | 0.33–7.02 | 2.2:1 |
| hsa-miR-188-5p | OR | 1.5 | 0.25–7.9 | 2.2:1 |
| hsa-miR-587 | OR | 1.8 | 0.12–25.15 | 2.2:1 |
| hsa-miR-490-5p | OR | 1.79 | 0.14–28.04 | 2.2:1 |
| hsa-miR-449b-3p | OR | 1.82 | 0.13–25.55 | 2.2:1 |
| hsa-miR-526b-5p | OR | 1.83 | 0.13–26.61 | 2.2:1 |
| hsa-miR-27a-3p | FC | 1.31 | 0.41–4.07 | 2.2:1 |
| hsa-miR-32-5p | OR | 1.85 | 0.14–31.41 | 2.1:1 |
| hsa-let-7g-5p | FC | 0.73 | 0.21–2.43 | 2.1:1 |
| hsa-miR-200c-3p | FC | 1.29 | 0.43–3.58 | 2.1:1 |
| hsa-miR-30b-5p | FC | 0.74 | 0.22–2.69 | 2.1:1 |
| hsa-miR-208a | OR | 1.81 | 0.13–27.72 | 2.1:1 |
| hsa-let-7i-3p | OR | 1.79 | 0.15–28.06 | 2.1:1 |
| hsa-miR-548m | OR | 0.59 | 0.06–5.01 | 2.1:1 |
| hsa-miR-1911-5p | OR | 1.81 | 0.14–31.41 | 2.1:1 |
| hsa-miR-554 | OR | 1.83 | 0.13–27.63 | 2.1:1 |
| hsa-miR-323b-5p | FC | 1.34 | 0.36–4.68 | 2.1:1 |
| hsa-miR-361-3p | FC | 1.33 | 0.39–4.62 | 2.1:1 |
| hsa-miR-1253 | OR | 1.8 | 0.13–29.94 | 2.1:1 |
| hsa-miR-325 | OR | 1.83 | 0.12–23.73 | 2.1:1 |
| hsa-miR-509-3-5p | OR | 1.81 | 0.15–28.39 | 2.1:1 |
| hsa-miR-802 | OR | 1.83 | 0.15–29.07 | 2.1:1 |
| hsa-miR-30d-5p | FC | 0.76 | 0.25–2.55 | 2.1:1 |
| hsa-miR-663b | FC | 0.72 | 0.18–3.07 | 2.1:1 |
| hsa-miR-548l | OR | 1.82 | 0.12–27.15 | 2.1:1 |
| hsa-miR-106b-3p | OR | 1.45 | 0.25–7.91 | 2.1:1 |
| hsa-miR-549 | OR | 1.82 | 0.11–24.88 | 2.1:1 |
| hsa-miR-214-5p | OR | 1.75 | 0.12–25.03 | 2.1:1 |
| hsa-miR-184 | OR | 1.54 | 0.2–11.1 | 2:01 |

CI 95, 95% confidence intervals; POR, posterior odds ratio; FC, fold change; OR, odd ratio.

| | | | | |
|---|---|---|---|---|
| hsa-miR-526b-3p | OR | 1.78 | 0.11–24.75 | 2:01 |
| hsa-miR-126-3p | OR | 0.71 | 0.15–3.22 | 2:01 |
| hsa-miR-937 | FC | 0.77 | 0.26–2.32 | 2:01 |
| hsa-miR-373-3p | OR | 0.6 | 0.06–5.27 | 2:01 |
| hsa-miR-191-5p | FC | 1.21 | 0.49–2.8 | 2:01 |
| hsa-miR-766-3p | OR | 1.39 | 0.3–6.07 | 2:01 |
| hsa-miR-628-3p | OR | 0.68 | 0.12–4.16 | 2:01 |
| hsa-miR-640 | OR | 0.72 | 0.16–3.48 | 1.9:1 |
| hsa-miR-664-3p | OR | 1.38 | 0.31–6.36 | 1.9:1 |
| hsa-miR-331-3p | OR | 1.38 | 0.31–7.39 | 1.9:1 |
| hsa-miR-29a-5p | OR | 0.71 | 0.16–3.12 | 1.9:1 |
| hsa-miR-345-5p | OR | 0.61 | 0.07–5.83 | 1.9:1 |
| hsa-miR-598 | FC | 0.8 | 0.28–2.51 | 1.9:1 |
| hsa-miR-744-5p | OR | 1.38 | 0.28–6.3 | 1.9:1 |
| hsa-miR-429 | FC | 1.28 | 0.39–4.66 | 1.9:1 |
| hsa-miR-2110 | FC | 0.8 | 0.27–2.26 | 1.9:1 |
| hsa-miR-593-3p | OR | 1.36 | 0.31–6.77 | 1.9:1 |
| hsa-miR-20a-5p | FC | 0.77 | 0.22–2.65 | 1.9:1 |
| hsa-miR-183-5p | FC | 0.79 | 0.23–2.59 | 1.8:1 |
| hsa-miR-671-5p | FC | 1.24 | 0.43–3.55 | 1.8:1 |
| hsa-miR-631 | OR | 1.35 | 0.28–5.81 | 1.8:1 |
| hsa-miR-720 | FC | 1.22 | 0.41–3.46 | 1.8:1 |
| hsa-miR-122-3p | OR | 1.37 | 0.24–7.5 | 1.8:1 |
| hsa-miR-590-5p | OR | 0.72 | 0.12–3.99 | 1.8:1 |
| hsa-miR-143-5p | OR | 0.71 | 0.12–4.26 | 1.8:1 |
| hsa-miR-34a-5p | OR | 0.74 | 0.16–3.61 | 1.8:1 |
| hsa-miR-1471 | FC | 0.83 | 0.28–2.46 | 1.8:1 |
| hsa-miR-129-2-3p | OR | 0.67 | 0.08–5.54 | 1.8:1 |
| hsa-miR-548j | OR | 0.66 | 0.08–6.43 | 1.8:1 |
| hsa-miR-338-3p | OR | 1.39 | 0.21–9.76 | 1.8:1 |
| hsa-miR-1244 | FC | 0.8 | 0.23–2.54 | 1.8:1 |
| hsa-miR-28-5p | OR | 0.73 | 0.14–3.52 | 1.8:1 |
| hsa-miR-1249 | OR | 1.39 | 0.19–9.9 | 1.8:1 |
| hsa-miR-564 | FC | 0.83 | 0.26–2.29 | 1.8:1 |
| hsa-miR-326 | OR | 0.73 | 0.11–4.17 | 1.7:1 |
| hsa-miR-378a-5p | OR | 0.67 | 0.07–5.76 | 1.7:1 |
| hsa-miR-10b-3p | OR | 0.71 | 0.11–4.76 | 1.7:1 |
| hsa-miR-532-3p | FC | 0.79 | 0.19–2.95 | 1.7:1 |
| hsa-miR-887 | OR | 1.29 | 0.29–5.73 | 1.7:1 |
| hsa-miR-1208 | OR | 1.37 | 0.23–10.25 | 1.7:1 |
| hsa-miR-100-5p | FC | 1.23 | 0.39–4.07 | 1.7:1 |
| hsa-miR-1237 | OR | 0.72 | 0.12–4.26 | 1.7:1 |
| hsa-miR-181a-2-3p | OR | 1.32 | 0.25–6.24 | 1.7:1 |
| hsa-miR-509-3p | FC | 1.21 | 0.37–3.63 | 1.7:1 |
| hsa-miR-148a-5p | OR | 0.67 | 0.08–6.13 | 1.7:1 |
| hsa-miR-371a-5p | OR | 1.36 | 0.2–8.99 | 1.7:1 |
| hsa-miR-551a | OR | 1.29 | 0.27–6.12 | 1.7:1 |
| hsa-miR-192-3p | OR | 0.67 | 0.07–5.45 | 1.7:1 |
| hsa-miR-148a-3p | FC | 0.8 | 0.19–3.26 | 1.7:1 |
| hsa-miR-518a-3p | OR | 0.67 | 0.07–5.36 | 1.7:1 |

CI 95, 95% confidence intervals; POR, posterior odds ratio; FC, fold change; OR, odd ratio.

| | | | | |
|---|---|---|---|---|
| hsa-miR-1266 | FC | 0.84 | 0.3–2.55 | 1.7:1 |
| hsa-miR-29b-1-5p | FC | 1.19 | 0.44–3.69 | 1.7:1 |
| hsa-miR-151a-5p | FC | 1.2 | 0.34–4.02 | 1.7:1 |
| hsa-miR-185-3p | FC | 1.22 | 0.34–4.44 | 1.6:1 |
| hsa-miR-196a-5p | FC | 1.22 | 0.33–4.3 | 1.6:1 |
| hsa-miR-222-3p | FC | 0.84 | 0.27–3.07 | 1.6:1 |
| hsa-miR-302a-3p | OR | 1.26 | 0.26–5.35 | 1.6:1 |
| hsa-miR-99a-5p | FC | 0.85 | 0.27–2.65 | 1.6:1 |
| hsa-miR-335-5p | OR | 0.8 | 0.16–3.53 | 1.6:1 |
| hsa-miR-25-3p | FC | 0.84 | 0.25–2.71 | 1.6:1 |
| hsa-miR-135a-5p | OR | 0.8 | 0.17–3.71 | 1.6:1 |
| hsa-miR-200b-5p | FC | 0.84 | 0.25–3.12 | 1.5:1 |
| hsa-miR-519d | OR | 0.72 | 0.08–6.42 | 1.5:1 |
| hsa-miR-125b-5p | FC | 0.86 | 0.29–2.88 | 1.5:1 |
| hsa-miR-124-3p | FC | 1.18 | 0.33–4 | 1.5:1 |
| hsa-miR-95 | FC | 1.21 | 0.29–4.85 | 1.5:1 |
| hsa-miR-103a-2-5p | OR | 0.72 | 0.08–6.92 | 1.5:1 |
| hsa-miR-33a-5p | FC | 1.14 | 0.37–3.38 | 1.5:1 |
| hsa-miR-30a-5p | FC | 0.86 | 0.28–2.65 | 1.5:1 |
| hsa-miR-510 | OR | 1.2 | 0.26–5.78 | 1.5:1 |
| hsa-miR-185-5p | FC | 0.85 | 0.21–2.98 | 1.5:1 |
| hsa-miR-518f-3p | OR | 0.74 | 0.07–7.46 | 1.5:1 |
| hsa-miR-141-5p | FC | 0.88 | 0.32–2.59 | 1.5:1 |
| hsa-miR-9-5p | FC | 0.85 | 0.23–3.34 | 1.4:1 |
| hsa-miR-562 | FC | 1.12 | 0.39–3.26 | 1.4:1 |
| hsa-miR-149-3p | FC | 0.86 | 0.24–3.14 | 1.4:1 |
| hsa-miR-708-3p | FC | 0.88 | 0.26–2.81 | 1.4:1 |
| hsa-let-7f-5p | FC | 0.88 | 0.26–2.84 | 1.4:1 |
| hsa-miR-146a-5p | FC | 0.87 | 0.22–3.5 | 1.4:1 |
| hsa-miR-193a-3p | OR | 0.76 | 0.08–8.44 | 1.4:1 |
| hsa-miR-520a-5p | OR | 0.77 | 0.08–9.07 | 1.4:1 |
| hsa-miR-148b-3p | OR | 1.17 | 0.21–6.42 | 1.4:1 |
| hsa-miR-643 | OR | 1.19 | 0.2–5.91 | 1.4:1 |
| hsa-miR-25-5p | OR | 1.21 | 0.11–13.7 | 1.4:1 |
| hsa-miR-204-5p | FC | 0.89 | 0.3–2.76 | 1.3:1 |
| hsa-miR-484 | FC | 0.91 | 0.31–2.74 | 1.3:1 |
| hsa-miR-31-5p | FC | 1.13 | 0.35–3.76 | 1.3:1 |
| hsa-miR-497-5p | OR | 0.85 | 0.16–4.68 | 1.3:1 |
| hsa-miR-221-3p | FC | 0.89 | 0.22–3.22 | 1.3:1 |
| hsa-miR-659-3p | OR | 1.16 | 0.11–13.58 | 1.3:1 |
| hsa-miR-138-5p | OR | 0.86 | 0.17–4.89 | 1.3:1 |
| hsa-miR-450a-5p | OR | 0.87 | 0.17–4.42 | 1.3:1 |
| hsa-miR-29a-3p | FC | 0.89 | 0.21–3.69 | 1.3:1 |
| hsa-miR-103b | FC | 1.1 | 0.26–4.09 | 1.3:1 |
| hsa-miR-873-5p | OR | 1.18 | 0.11–14.08 | 1.3:1 |
| hsa-miR-93-3p | OR | 1.13 | 0.19–5.36 | 1.3:1 |
| hsa-miR-132-3p | FC | 1.09 | 0.35–3.16 | 1.3:1 |
| hsa-miR-760 | FC | 0.91 | 0.29–2.88 | 1.3:1 |
| hsa-miR-361-5p | OR | 1.17 | 0.11–12.84 | 1.3:1 |
| hsa-miR-212-3p | FC | 1.11 | 0.32–4.37 | 1.3:1 |

CI 95, 95% confidence intervals; POR, posterior odds ratio; FC, fold change; OR, odd ratio.

| | | | | |
|---|---|---|---|---|
| hsa-miR-221-5p | OR | 1.12 | 0.22–4.97 | 1.3:1 |
| hsa-miR-320b | FC | 1.08 | 0.35–2.99 | 1.2:1 |
| hsa-let-7i-5p | FC | 1.11 | 0.28–4.39 | 1.2:1 |
| hsa-miR-138-2-3p | OR | 1.16 | 0.11–11.99 | 1.2:1 |
| hsa-miR-449b-5p | FC | 1.1 | 0.24–4.84 | 1.2:1 |
| hsa-miR-106a-5p | FC | 0.92 | 0.25–3.14 | 1.2:1 |
| hsa-miR-297 | FC | 0.94 | 0.35–2.94 | 1.2:1 |
| hsa-miR-152 | FC | 0.93 | 0.24–3.79 | 1.2:1 |
| hsa-miR-216b | OR | 0.88 | 0.14–5.72 | 1.2:1 |
| hsa-miR-744-3p | FC | 1.07 | 0.37–3.18 | 1.2:1 |
| hsa-miR-224-3p | OR | 1.07 | 0.2–5.27 | 1.2:1 |
| hsa-miR-517c-3p | OR | 0.88 | 0.15–5.37 | 1.2:1 |
| hsa-miR-101-3p | FC | 1.1 | 0.22–6.32 | 1.2:1 |
| hsa-miR-576-3p | OR | 1.09 | 0.1–10.1 | 1.2:1 |
| hsa-miR-520d-3p | FC | 0.93 | 0.22–3.28 | 1.2:1 |
| hsa-miR-149-5p | FC | 0.95 | 0.31–3.06 | 1.2:1 |
| hsa-miR-24-1-5p | OR | 1.07 | 0.11–12.44 | 1.2:1 |
| hsa-miR-525-5p | OR | 1.07 | 0.09–9.47 | 1.2:1 |
| hsa-miR-1911-3p | OR | 1.05 | 0.17–7.36 | 1.2:1 |
| hsa-miR-609 | OR | 1.08 | 0.1–11.03 | 1.2:1 |
| hsa-miR-508-5p | OR | 1.07 | 0.11–10.76 | 1.2:1 |
| hsa-miR-877-3p | FC | 0.95 | 0.31–2.84 | 1.2:1 |
| hsa-miR-28-3p | FC | 1.05 | 0.33–3.15 | 1.2:1 |
| hsa-miR-16-2-3p | OR | 1.07 | 0.1–11.41 | 1.1:1 |
| hsa-miR-383 | OR | 1.06 | 0.11–11.51 | 1.1:1 |
| hsa-miR-125a-3p | OR | 1.05 | 0.1–11.22 | 1.1:1 |
| hsa-miR-493-5p | OR | 1.08 | 0.11–10.76 | 1.1:1 |
| hsa-miR-129-5p | OR | 1.06 | 0.11–9.93 | 1.1:1 |
| hsa-miR-646 | OR | 0.93 | 0.17–6.28 | 1.1:1 |
| hsa-miR-1258 | OR | 1.07 | 0.11–11.42 | 1.1:1 |
| hsa-miR-875-3p | OR | 1.04 | 0.1–11.07 | 1.1:1 |
| hsa-miR-423-3p | FC | 0.94 | 0.26–3.34 | 1.1:1 |
| hsa-miR-671-3p | OR | 1.08 | 0.11–11.6 | 1.1:1 |
| hsa-miR-629-3p | FC | 1.03 | 0.29–3.62 | 1.1:1 |
| hsa-miR-513c-5p | OR | 1.05 | 0.1–10.75 | 1.1:1 |
| hsa-miR-501-5p | FC | 1.06 | 0.26–4.18 | 1.1:1 |
| hsa-miR-922 | OR | 1.06 | 0.11–10.83 | 1.1:1 |
| hsa-miR-181a-3p | OR | 1.04 | 0.1–10.74 | 1.1:1 |
| hsa-miR-499a-5p | OR | 1.04 | 0.11–10.83 | 1.1:1 |
| hsa-miR-92a-2-5p | OR | 1.04 | 0.09–9.56 | 1.1:1 |
| hsa-miR-30c-5p | FC | 0.97 | 0.32–3.19 | 1.1:1 |
| hsa-miR-18a-3p | OR | 1.06 | 0.11–11.15 | 1.1:1 |
| hsa-miR-532-5p | FC | 0.96 | 0.22–3.6 | 1.1:1 |
| hsa-miR-196b-5p | OR | 0.96 | 0.22–4.84 | 1.1:1 |
| hsa-miR-517a-3p | OR | 0.92 | 0.11–9.51 | 1.1:1 |
| hsa-miR-193b-3p | FC | 1.04 | 0.26–4.18 | 1.1:1 |
| hsa-miR-30a-3p | FC | 1.03 | 0.35–3.12 | 1.1:1 |
| hsa-miR-668 | OR | 1.04 | 0.1–10.02 | 1.1:1 |
| hsa-miR-525-3p | OR | 0.92 | 0.1–9.52 | 1.1:1 |
| hsa-miR-338-5p | OR | 1.02 | 0.11–11.16 | 1.1:1 |

CI 95, 95% confidence intervals; POR, posterior odds ratio; FC, fold change; OR, odd ratio.

| | | | | |
|---|---|---|---|---|
| hsa-miR-144-5p | OR | 0.92 | 0.1–9.01 | 1.1:1 |
| hsa-miR-15a-5p | FC | 1.05 | 0.26–4.14 | 1.1:1 |
| hsa-miR-514a-3p | OR | 0.94 | 0.16–6.05 | 1.1:1 |
| hsa-miR-550a-3p | OR | 0.95 | 0.16–5.83 | 1.1:1 |
| hsa-miR-103a-3p | FC | 0.97 | 0.35–2.43 | 1.1:1 |
| hsa-miR-301a-3p | OR | 0.96 | 0.15–5.35 | 1.1:1 |
| hsa-miR-92a-1-5p | OR | 1.03 | 0.11–11.13 | 1.1:1 |
| hsa-miR-19a-3p | FC | 1.03 | 0.24–4.83 | 1:01 |
| hsa-miR-623 | OR | 0.95 | 0.15–5.74 | 1:01 |
| hsa-miR-580 | OR | 0.92 | 0.1–9.84 | 1:01 |
| hsa-miR-596 | OR | 1 | 0.14–5.61 | 1:01 |
| hsa-miR-134 | FC | 1.02 | 0.34–2.95 | 1:01 |
| hsa-miR-595 | FC | 1.01 | 0.32–2.84 | 1:01 |
| hsa-miR-652-3p | OR | 0.97 | 0.18–5.34 | 1:01 |
| hsa-miR-625-3p | OR | 0.94 | 0.1–9.3 | 1:01 |
| hsa-miR-487a | OR | 0.94 | 0.1–9.86 | 1:01 |
| hsa-let-7f-1-3p | OR | 0.95 | 0.1–9.15 | 1:01 |
| hsa-miR-181a-5p | FC | 0.99 | 0.26–3.54 | 1:01 |
| hsa-miR-298 | OR | 0.94 | 0.08–8.49 | 1:01 |
| hsa-miR-24-3p | FC | 0.99 | 0.33–3.08 | 1:01 |
| hsa-miR-512-5p | OR | 0.95 | 0.1–9.48 | 1:01 |
| hsa-miR-372 | OR | 0.96 | 0.09–8.99 | 1:01 |
| hsa-miR-147b | OR | 0.96 | 0.11–10.56 | 1:01 |
| hsa-let-7b-5p | FC | 0.99 | 0.32–2.9 | 1:01 |
| hsa-miR-181b-5p | OR | 0.99 | 0.22–4.24 | 1:01 |

CI 95, 95% confidence intervals; POR, posterior odds ratio; FC, fold change; OR, odd ratio.
